# The uncharacterized PA3040-3042 operon is part of the cell envelope stress response and a tobramycin resistance determinant in a clinical isolate of *Pseudomonas aeruginosa*

**DOI:** 10.1128/spectrum.03875-23

**Published:** 2024-07-01

**Authors:** Magnus Z. Østergaard, Flemming D. Nielsen, Mette H. Meinfeldt, Clare L. Kirkpatrick

**Affiliations:** 1 Department of Biochemistry and Molecular Biology, University of Southern Denmark, Odense, Denmark; 2 Department of Clinical Microbiology, Odense University Hospital, Odense, Denmark; Centre National de la Recherche Scientifique, Marseille, France

**Keywords:** bacteriophage, transposon insertion sequencing, biofilm

## Abstract

Bacteriophages (hereafter “phages”) are ubiquitous predators of bacteria in the natural world, but interest is growing in their development into antibacterial therapy as complement or replacement for antibiotics. However, bacteria have evolved a huge variety of antiphage defense systems allowing them to resist phage lysis to a greater or lesser extent. In addition to dedicated phage defense systems, some aspects of the general stress response also impact phage susceptibility, but the details of this are not well known. In order to elucidate these factors in the opportunistic pathogen *Pseudomonas aeruginosa*, we used the laboratory-conditioned strain PAO1 as host for phage infection experiments as it is naturally poor in dedicated phage defense systems. Screening by transposon insertion sequencing indicated that the uncharacterized operon PA3040-PA3042 was potentially associated with resistance to lytic phages. However, we found that its primary role appeared to be in regulating biofilm formation, particularly in a clinical isolate of *P. aeruginosa* in which it also altered tobramycin resistance. Its expression was highly growth-phase dependent and responsive to phage infection and cell envelope stress. Our results suggest that this operon may be a cryptic but important locus for *P. aeruginosa* stress tolerance.

**IMPORTANCE:**

An important category of bacterial stress response systems is bacteriophage defense, where systems are triggered by bacteriophage infection and activate a response which may either destroy the phage genome or destroy the infected cell so that the rest of the population survives. In some bacteria, the cell envelope stress response is activated by bacteriophage infection, but it is unknown whether this contributes to the survival of the infection. We have found that a conserved uncharacterized operon (PA3040-PA3042) of the cell envelope stress regulon in *Pseudomonas aeruginosa*, which has very few dedicated phage defense systems, responds to phage infection and stationary phase as well as envelope stress and is important for growth and biofilm formation in a clinical isolate of *P. aeruginosa*, even in the absence of phages. As homologs of these genes are found in other bacteria, they may be a novel component of the general stress response.

## INTRODUCTION

*Pseudomonas aeruginosa* is an environmentally ubiquitous bacterium that is an opportunistic pathogen of humans and animals. It is naturally tolerant to antibiotics which, in conjunction with its highly adaptable metabolism and stress response systems, establish it as a notoriously resilient pathogen (1). These features are particularly problematic in the ongoing antibiotic resistance crisis. *P. aeruginosa* can cause a wide range of infections in susceptible individuals, including burn wound infections, sepsis, and pneumonia (2). The latter is of particular note in cystic fibrosis, where *P. aeruginosa* establishes as a chronic, stress-tolerant biofilm capable of evading the host immune system and tolerating antibiotics. While antibiotic treatment can alleviate symptoms (3), chronic, biofilm-associated *P. aeruginosa* infections are near-impossible to eradicate (4). Phage therapy has been suggested as an alternative or complement to such biofilm-associated infections, as they have several advantages over antibiotics in that particular niche. The biofilm matrix itself can restrict the diffusion of antibiotics, which is also less effective on the less metabolically active cells. Phage diffusion can be restricted by biofilm (5) but can counter with encoded depolymerases that disperse biofilm (6). While neither phage nor antibiotic can effectively kill dormant cells, the phage can lie dormant as a pseudolysogen and eventually resume the lytic cycle when host metabolic activity resumes (7).

Bacteriophage defense systems are ubiquitous among bacteria but vary greatly in mechanism and specificity among species and strains. Classical examples include sequence-based restriction of phage DNA through restriction/modification (R/M) systems or acquired sequence-based restriction in CRISPR-Cas. These restrict foreign DNA with little cost to the infected bacterium, in contrast to “altruistic” abortive infection (Abi), where an infected bacterium dies without producing phage progeny, sacrificing itself to protect the population at large (8). Current high-throughput discovery strategies predict phage defense loci based on their presence in “defense islands”—genomic loci that harbor multiple phage defense systems of varied mechanisms. This enables the discovery of novel defense systems by their association with known ones. This has led to the discovery of a multitude of phage defense systems (9). Recently, a screening of numerous *P. aeruginosa* strains has revealed two core defense hotspots in the *P. aeruginosa* pangenome. R/M systems are the most common in both hotspots, which also display remarkable diversity (10). A disadvantage of this approach is its prediction-based nature which is dependent on input data. In addition, defense islands tend to contain dedicated phage defense systems, meaning that multifunctional genes with a secondary effect on phage defense are likely in other loci. In addition to dedicated phage defense systems found in distinct loci, core parts of *P. aeruginosa* biology also provide some measure of phage defense, though this is arguably secondary to the main biological function of environment sensing and response. Examples include quorum sensing-mediated swarming repulsion from infected cells (11), biofilm exopolysaccharides protecting aggregated cells from phage (5), and induction of CRISPR-Cas by quorum sensing of phage attack (12).

Biofilm exopolysaccharides are also modulated during the cell envelope stress response, a protective mechanism that is induced when the integrity of the cell wall is perturbed by external factors such as osmotic stress, cell envelope-targeted antibiotics, or interaction with the immune system during infection. In *P. aeruginosa*, these exopolysaccharides are Psl, Pel, and alginate, the latter of which is directly controlled by cell envelope stress-responsive sigma factor AlgU (also known as AlgT or σ^22^) (1, 13). Though the association between cell envelope stress and alginate biosynthesis is not fully elucidated, alginate overproduction or “mucoid conversion” can be conferred through mutation of AlgU’s cognate anti-sigma factor MucA, which is often seen in mucoid isolates from chronic infections in CF patients (13, 14). D-cycloserine, a cell envelope-stressing antibiotic commonly used to study AlgU, does not cause mucoid conversion despite leading to MucA degradation (15). Alginate is not required for biofilm formation in non-mucoid strains, which can synthesize Pel and/or Psl in a strain-dependent manner. The reference strain PAO1 primarily synthesizes Psl, whereas the model virulent strain PA14 exclusively synthesizes Pel (16). Although production of these exopolysaccharides has not been found to be directly related to cell envelope stress response, deletion of *algU* and *sigX* (another major cell envelope stress-responsive sigma factor in *P. aeruginosa*) abrogates Psl- and Pel-mediated biofilm formation, respectively, in PAO1, indicating that the cell envelope stress response indirectly regulates biofilm formation (17, 18).

A valuable forward genetics option for discovery of stress tolerance mechanisms not requiring *a priori* assumptions is transposon sequencing (Tn-Seq). Here, challenging a transposon mutant library with a stressor, such as phage infection, can be viewed as thousands of competition experiments performed in parallel, where mutants with altered susceptibility can be identified (19). High-throughput sequencing is used to recover and quantify transposon junction reads, which are used to identify insertion sites and as a proxy for mutant fitness. This has been applied to discover both phage susceptibility genes/phage receptors and phage defense genes in other species (20–22). In this context, loss of function of a susceptibility gene by transposon insertion would confer a selective advantage on those cells, leading to enrichment of that transposon junction read in phage-infected conditions relative to control. By contrast, transposon insertions into loci, which contribute to phage defense, should lead to depletion of those transposon junction reads in phage-infected conditions relative to control since they would be at a selective disadvantage for proliferation in the presence of phages, regardless of whether they have inactivated a dedicated phage defense system or an alternative system which contributes to phage tolerance as a secondary effect.

Here, we employed Tn-Seq to investigate the presence of alternative loci which might contribute to phage survival in *P. aeruginosa* PAO1, a lab-conditioned strain which has a very well annotated genome with notably few dedicated phage defense systems (23). Our screen identified a range of loci, including one that has been implicated in responses to phage infection in other studies. Further study of a selected locus, the PA3040-3042 operon, showed that it was transcriptionally responsive to cell envelope stress and to phage exposure, although deletion of the operon did not affect tolerance of phage exposure as would have been expected for a dedicated phage defense system. Instead, we observed that it was highly expressed in stationary phase planktonic cells, that high-level overexpression of the whole operon reduced biofilm formation, and that deletion of the operon or the final gene PA3042 caused growth and biofilm defects and altered tobramycin resistance in a clinical isolate of *P. aeruginosa*. Our results point to this operon as an important subject for further study in the context of *P. aeruginosa* stress responses, particularly cell envelope stress.

## RESULTS

### Genome-wide unbiased screening of the laboratory-conditioned *Pseudomonas aeruginosa* strain PAO1 indicates novel loci implicated in bacteriophage survival

**Fig 1:**
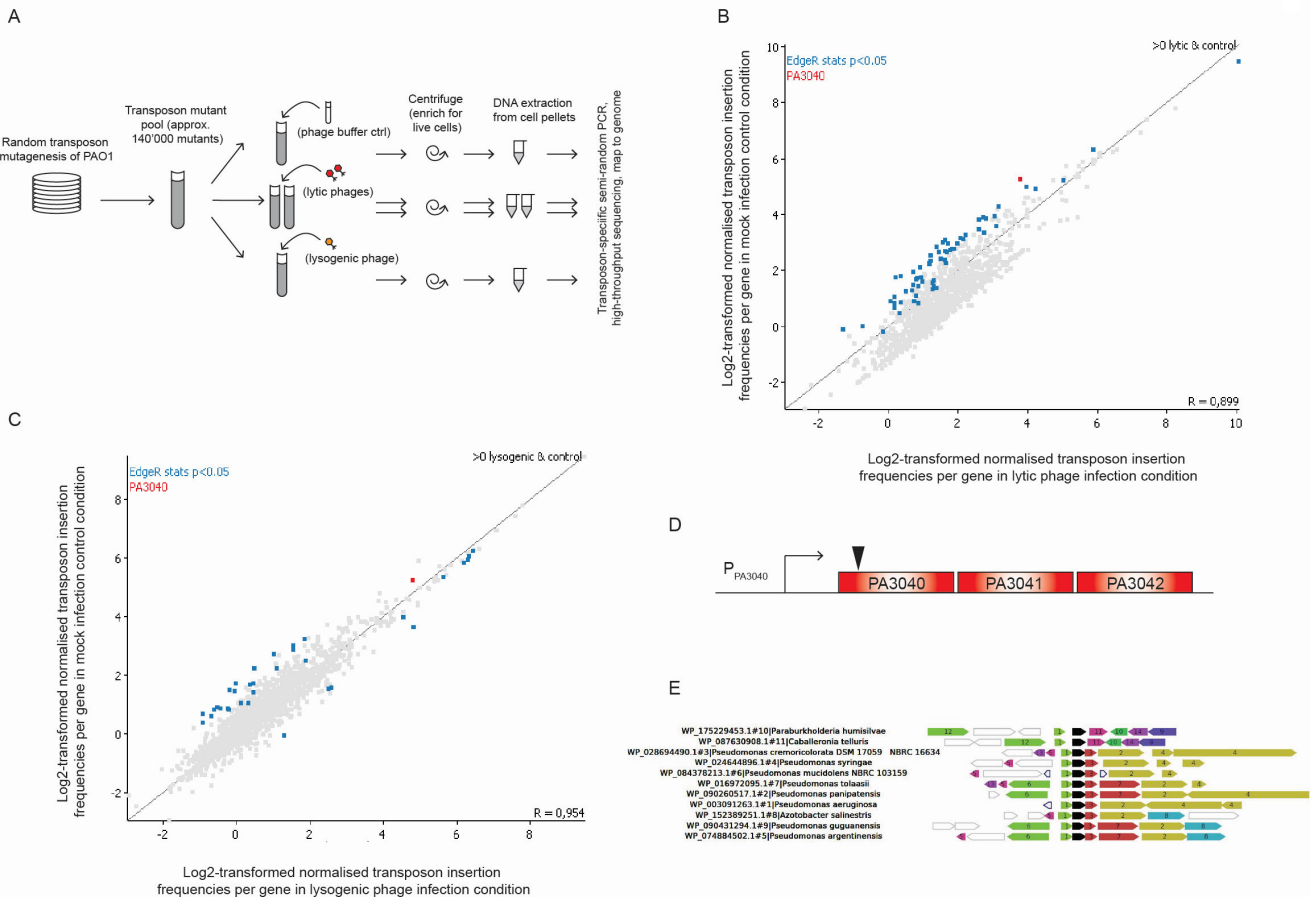
Transposon insertions in the PA3040 gene are among the most depleted insertions in lytic phage infection conditions relative to the control. (**A**) Workflow of the transposon insertion sequencing experiment. (**B**) Scatter plot of the normalized data from averages of the three biological replicates of both lytic phages combined. Every point represents normalized transposon insertions into one gene. Blue points are those where insertions into those genes are statistically significantly depleted in the phage infection conditions relative to mock infection. The point representing transposon insertions into PA3040 (also *P* < 0.05) is highlighted in red. Note that the data normalization involves correcting for total read count in all libraries, and since many fewer reads were recovered from the lytic phage-treated cultures than from the controls, the diagonal line of unchanged data points is shifted right relative to the *y* = *x* diagonal line that is plotted automatically on the graph. (**C**) Scatter plot of the normalized data from averages of the three biological replicates of the lysogenic phage. Every point represents normalized transposon insertions into one gene. Blue points are those where insertions into those genes are statistically significantly depleted in the phage infection conditions relative to mock infection. The point representing transposon insertions into PA3040 is highlighted in red as in (**B**), although in this experiment it was not statistically different in lysogenic phage infection relative to control. (**D**) Arrangement of the PA3040-PA3042 operon. The triangle represents the position of the individual transposon insertion into PA3040 which was mapped from the sequencing data (approximately to scale). (**E**) The PA3040-PA3042 operon is conserved among *Pseudomonas* species and partially conserved among other gamma-proteobacteria genera as well. Analysis was performed using WebFlags with the translated PA3041 gene (in black) as the query and searched against the RefSeq protein database with the default settings of maximum 200 homologs in the alignment and a cutoff of *E* = 10^−3^. Note that some of the occurrences of PA3042 here are likely derived from more distantly related homologous proteins in the RefSeq database which did not occur in our more restricted data set used for pangenome construction and/or did not pass our sequence identity criteria for being the same gene. Gene product names, as generated by WebFlags (with corresponding PAO1 locus tag in parenthesis where relevant) are 1, YqjD family protein (PA3040); 2, deoxyguanosinetriphosphate triphosphohydrolase (PA3043); 3, hypothetical protein (PA3042); 4, DUF883 family protein (PA3044/PA3045); 5, response regulator; 6, deoxyguanosinetriphosphate triphosphohydrolase; 7, YqjD family protein; 8, glutaredoxin family protein; 9, ATP-binding protein; 10, *pca* operon transcription factor PcaQ; 11, acyltransferase; 12, hypothetical protein; 13, response regulator transcription factor; 14, ammonium transporter.

We hypothesized that using a model organism known to be poor in “classical” phage defense systems could be useful in uncovering novel genetic loci or stress response systems that contribute to the survival of phage infection. To assess the baseline defense system content of this strain, we used the tools Defense Finder (24) and Padloc (25) on the *P. aeruginosa* PAO1 genome (accession number NC_002516.2). This returned the genes PA1371-1372 (helicases), PA2732 and PA2735 (components of an R/M system), PA0715-0716 and the sRNA *phrD* (retron), and PA1939 (putative member of the Gabija family of novel phage defense systems [9]) as hits, confirming that this strain does indeed have a very small number of classical phage defense systems. Therefore, to investigate whether we could identify other loci which contributed to the survival of phage infection, we designed a Tn-Seq infection screen using PAO1 as host strain with three newly isolated phages, two lytic and one lysogenic. We generated a transposon library of approximately 140,000 independent mutants, passaged it twice through fresh medium to enrich for fully viable cells, and challenged it with infection at MOI = 10 of the three phages alongside a mock infection control (Fig. 1A). After total DNA extraction from the cell pellet, recovered transposon insertions from each condition were mapped against the PAO1 genome, and normalized transposon insertions per gene were plotted for phage infection against mock infection. The resulting scatter plots (Fig. 1B shows the combined data for both lytic phages against mock infection control since both were highly similar, and Fig. 1C shows the combined data for the lysogenic phage) show that the data cluster around a diagonal correlation line, indicating no difference in viability of the majority of the transposon insertions between infection and control conditions but with some outliers on either side. Outliers that are proportionally enriched in control/depleted in phage infection conditions would indicate loci that contribute to survival of phage infection (if inactivating mutations in those genes lead to a greater degree of loss of those clones out of the pooled library when phages are present). This would primarily indicate loci which contribute to survival at the individual cell level since inactivating mutations in systems which contribute to survival at the population level (in a genetically homogeneous population) through death of phage-infected cells by abortive infection mechanisms would not show this profile in a competition experiment such as Tn-Seq. Transposon insertions in Abi systems would either be compensated for by other defense systems in the same cell that were not disrupted or would be neutral because the readout of the experiment is based on failure to proliferate without any way to distinguish cell death due to phage infection from cell death due to toxin activity.

Statistical analysis of the data set gave 64 genes, which were significantly (*P* < 0.05) changed in the lytic phage-infected samples (63 depleted/1 enriched) indicated in blue in Fig. 1B, and 33 genes, which were significantly changed in the lysogenic phage infected sample (23 depleted/10 enriched, Fig. 1C) (Data Set S1). Furthermore, 10 genes were common to both data sets. Among the shared genes depleted in phage infection was *spuE*, from a polyamine synthesis and export system, which has been previously implicated in phage defense (26). To investigate whether any particular functional categories of genes were associated with the significantly altered genes, we used clusters of orthologous genes (COG) category assignment. Here, we observed that the most over-represented categories were energy production and conversion (C), amino acid transport and metabolism (E), and cell wall/membrane/envelope biogenesis (M) (Table 1). We did not observe the previously mentioned defense system genes of PAO1 to be significantly depleted in any of our phage infection conditions relative to control or for the COG category of defense mechanisms (V) to be enriched among the significantly altered genes.

**TABLE 1:** Phage infection causes significant changes in Tn insertion frequency in genes assigned to functional categories of energy production and conversion, amino acid transport and metabolism, and cell wall/membrane/envelope biogenesis^*a*^

COG category	Lytic phage		Lysogenic phage	
	Count	Percentage	Count	Percentage
B: Chromatin structure/dynamics	0	0.0	0	0.0
C: Energy production/conversion	6	9.4	4	12.1
D: Cell cycle control, cell division, and chromosome partitioning	2	3.1	0	0.0
E: Amino acid transport/metabolism	7	10.9	4	12.1
F: Nucleotide transport/metabolism	1	1.6	2	6.1
G: Carbohydrate transport/metabolism	3	4.7	1	3.0
H: Coenzyme transport/metabolism	1	1.6	0	0.0
I: Lipid transport/metabolism	1	1.6	3	9.1
J: Translation, ribosomal structure, and biogenesis	1	1.6	0	0.0
K: Transcription	4	6.3	1	3.0
L: Replication, recombination, and repair	1	1.6	2	6.1
M: Cell wall/membrane/envelope biogenesis	6	9.4	7	21.2
N: Cell motility	1	1.6	0	0.0
O: Post-translational modification, protein turnover, chaperones	1	1.6	0	0.0
P: Inorganic ion transport/metabolism	3	4.7	1	3.0
Q: Secondary metabolites biosynthesis, transport, and catabolism	1	1.6	0	0.0
R: General function	0	0.0	0	0.0
S: Unknown	11	17.2	3	9.1
T: Signal transduction	4	6.3	3	9.1
U: Intracellular trafficking, secretion, and vesicular transport	2	3.1	0	0.0
V: Defense mechanisms	2	3.1	0	0.0
Unassigned or >1 category	6	9.4	2	6.1

^*a*^ COG category assignment counts for the differentially enriched/depleted Tn insertions into lytic or lysogenic phage infection conditions relative to mock-infected control. Counts are out of 64 for the lytic phage infection conditions and out of 33 for the lysogenic phage.

Since lytic phages hold the most promise for phage therapy, we focused particularly on the genes that were significantly depleted out of the lytic phage infection conditions but not lysogenic phage or control. Here, the gene PA3040 was one of the most prominent, with a substantial fold change between lytic phage infection and control (Fig. 1B) but not significantly changed between lysogenic phage infection and control, suggesting that it could be a candidate system for lytic phage survival. Closer inspection of the Tn-Seq data showed that Tn insertions were not well represented in this region, with one insertion close to the 5′ end of PA3040 accounting for all of the recovered reads. However, in the genomic context, PA3040 appears to be the first gene in a three-gene operon, with very close spacing (<15 bp) between the open reading frames of PA3040, PA3041, and PA3042 (Fig. 1D). It is therefore likely that Tn disruption in PA3040 would inactivate all three genes through polar effects.

### The PA3040-3042 operon is part of the *Pseudomonas aeruginosa* core genome

The functions of all three genes are experimentally undefined. At the sequence level, PA3040 has a DUF883 domain and is similar to *yqjD* from *E. coli*, a ribosome-binding membrane protein (27). PA3041 resembles *yqjE*, a phage holin homolog associated with growth inhibition when overexpressed (28), while PA3042 is a conserved hypothetical protein with no recognized domains. Analysis of genomic context conservation by webFlaGs (29) using PA3041 as query showed that paralogs of this gene are found among a large number of *Pseudomonas* species and some other gamma-proteobacterial species as well (a subset of the data selected to show different genomic contexts is shown in Fig. 1E; running the analysis with an unrestricted number of genomes led to an alignment of almost 200 sequences containing these genes). We then performed a more rigorous pangenome analysis at the species and genus level to define to what extent the conservation is maintained, using a stricter cutoff (*E* = 10^−5^) in order to identify only occurrences of the same gene (excluding paralogs with lower overall sequence identity). Using 640 *Pseudomonas aeruginosa* genomes to construct a species-level pangenome, we observed that this operon is part of the core genome of this species, as it was present in 100% of the genomes. All three genes were consistently found together, and the copy number was always one. The nucleotide sequence was also highly conserved (PA3040 99.79%, PA3041 99.70%, and PA3042 99.37%).

Extending the analysis to the genus level, we used a set of 113 selected representative genomes from the *Pseudomonas* genus (one per species), including the PAO1 genome as the sole representative of the *Pseudomonas aeruginosa* species (Table S1). Using the same stringent cutoff, we observed that the PA3040 and PA3041 genes were significantly more conserved across the genus than PA3042. PA3040 was found in 109/113 genomes and PA3041 in 106/113 genomes, but PA3042 was only found in 2 genomes: the PAO1 reference genome and in *Pseudomonas paraeruginosa* (a close relative of *P. aeruginosa* that includes the commonly used strain PA7*,* only recently defined as a separate species [30]). Hence, the PA3040-PA3042 operon is part of the *P. aeruginosa* species core genome but not of the *Pseudomonas* genus core genome. At genus level, the first two genes are conserved almost across the entire genus data set, but PA3042 is specific to *P. aeruginosa* and its closest relative, suggesting that it provides some key function to the *aeruginosa* species, which has mostly not been retained in other pseudomonads.

### The PA3040-3042 operon alters colony morphology but not growth rate in PAO1

**Fig 2:**
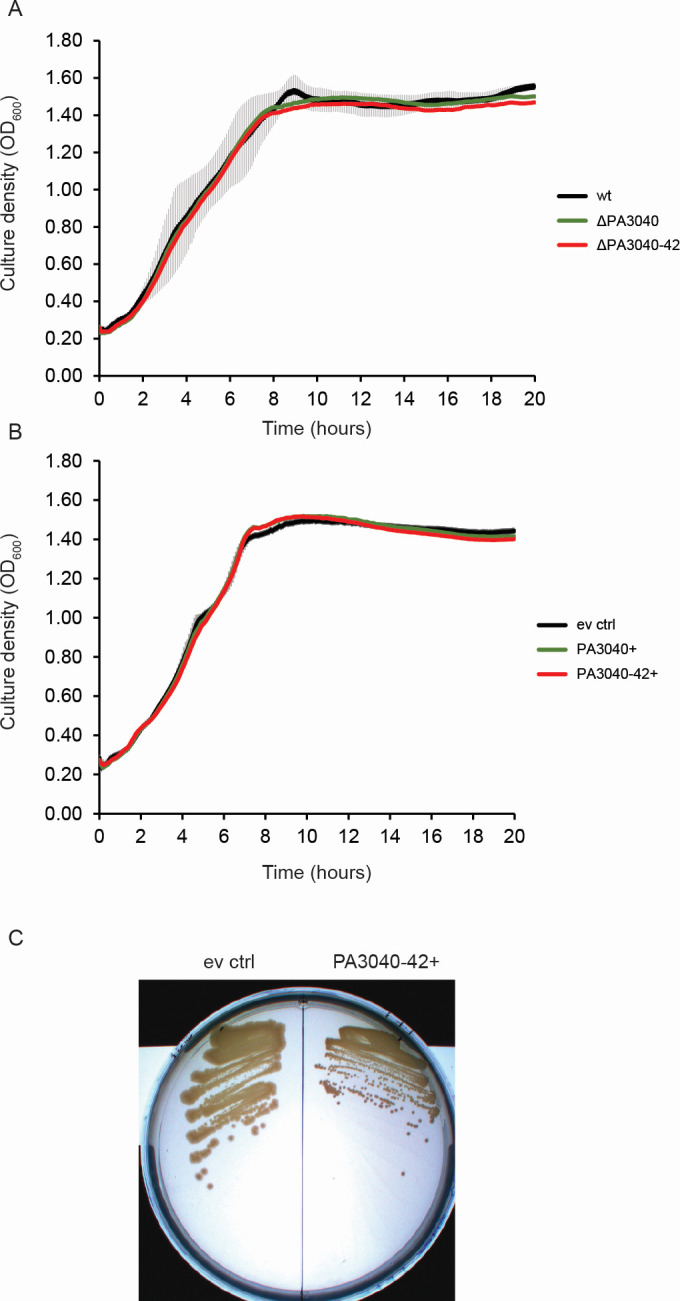
Overexpression of the PA3040-PA3042 operon inhibits biofilm formation without affecting growth rate. (**A**) Growth kinetics of WT PAO1 and deletion mutants of PA3040 or the whole operon. Error bars corresponding to the standard deviation of the biological replicates of the wild type only are shown because the error bar range for all data series was superimposable, and no significant difference in growth rates between any strains was observed. (**B**) Growth kinetics of WT PAO1 containing empty vector (ev) control (pSRK::Gent with no insert) or overexpression constructs of PA3040 or the PA3040-3042 operon in the same vector. All cultures were incubated in the presence of gentamicin to maintain selection on the plasmid and 1 mM IPTG to induce overexpression of the genes. Error bars for the empty vector control only are shown as in part **A**. (**C**) Colony morphology of WT PAO1 containing either empty vector pSRK::Gent or the overexpression plasmid for the PA3040-3042 operon (pSRK::PA3040-3042), after growth on LB medium with 1 mM IPTG for 16 hours.

Since paralogs of PA3040 and PA3041 had been implicated in growth inhibition of *E. coli* when overexpressed (27, 28), we constructed deletion and overexpression strains for the PA3040 gene and the whole PA3040-3042 operon to examine whether this was also the case in *Pseudomonas aeruginosa* PAO1. Neither deletion (Fig. 2A) nor overexpression (Fig. 2B) of the PA3040-3042 genes affected growth kinetics. However, we noted an effect of whole operon overexpression on colony morphology, with colonies appearing smaller, regularly shaped, and rough (Fig. 2C). PAO1 normally makes smooth, irregular edged, and relatively large colonies (Fig. 2C, left) but is not mucoid since it does not produce alginate during unstressed conditions (31). Overexpression of the individual genes did not affect colony morphology (not shown), suggesting that the whole operon is required to achieve this effect. Although the smaller colonies were suggestive of small colony variant (SCV) formation, the overexpression strain did not exhibit any other phenotypes associated with SCVs, which normally have a slower growth rate, overproduce extracellular polysaccharides and biofilm, and can show reduced motility. We conclude that the most likely explanation is that cell surface properties (possibly including extracellular polysaccharides) are altered by overexpression of PA3040-3042 but not in a way that converts the strain to SCV.

### The PA3040-3042 operon is transcriptionally induced by cell envelope stress, bacteriophage exposure, and stationary phase

**Fig 3:**
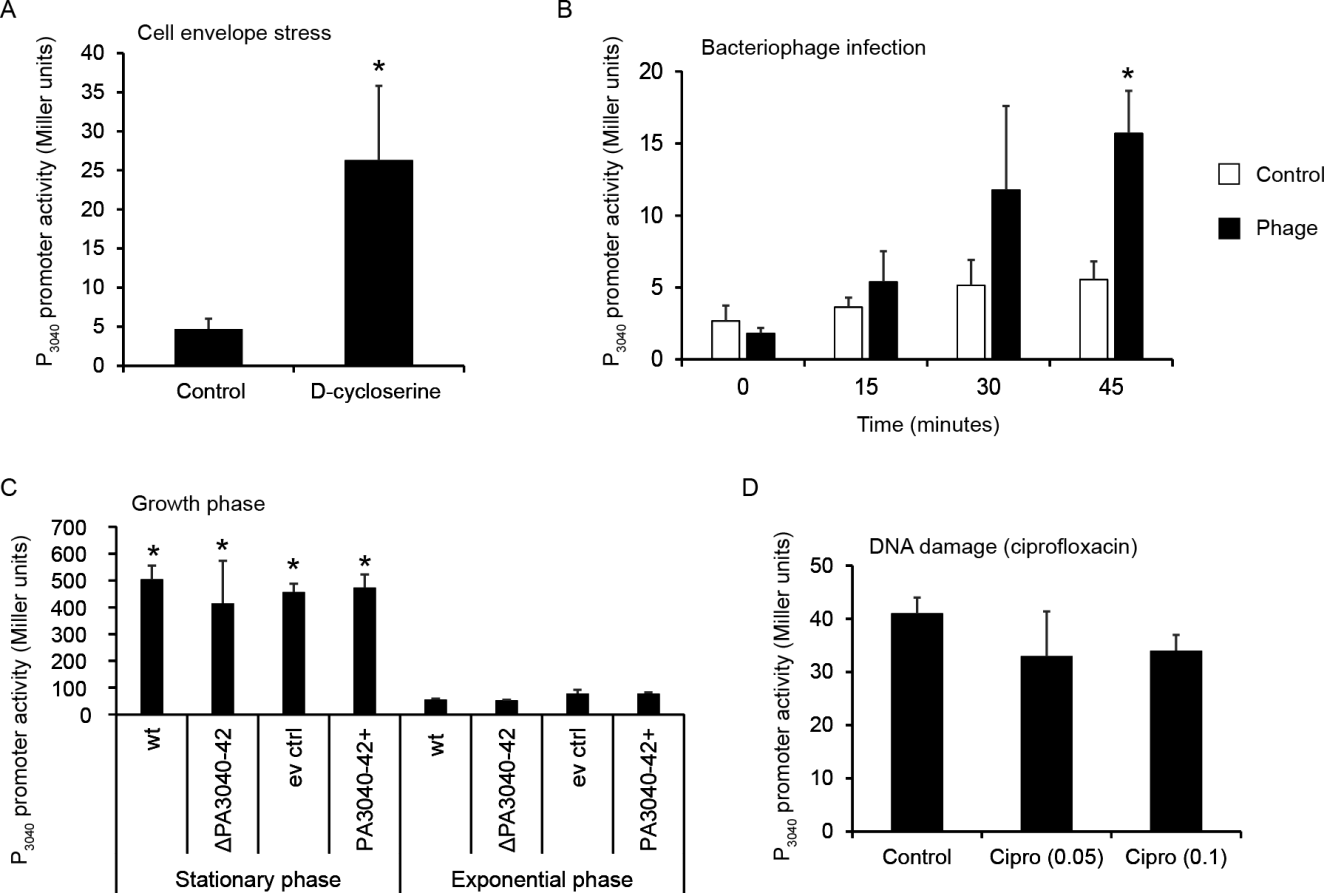
The promoter of the PA3040-3042 operon is induced by cell envelope stress, bacteriophage exposure, and stationary phase. Beta-galactosidase activity of a transcriptional fusion to the PA3040-3042 promoter under various stress conditions. Asterisks indicate statistically significant differences between test condition and matched control with *P* < 0.05. (**A**) Promoter activity in WT exponential phase cells with 800 µg/mL D-cycloserine. (**B**) Promoter activity in exponential phase WT cells during a time course of acute exposure to lytic phage ΦMSPA56 (MOI = 10) or phage buffer control sampled every 15 minutes for 45 minutes. Asterisk indicates statistically significant difference between 0 and 45 minutes for the phage-treated sample. (**C**) Promoter activity in WT, ΔPA3040-3042, WT containing pSRK::Gent empty vector, and WT containing pSRK::PA3040-3042, in cultures sampled in exponential phase at OD600 = 0.5 and in stationary phase at OD600 = 4. Cultures containing pSRK::Gent empty vector or pSRK::PA3040-3042 were supplemented with gentamicin and 1 mM IPTG. Asterisks indicate statistically significant differences between exponential and stationary phase activity within each strain. (**D**) Promoter activity in exponential phase WT cells with 0.05 or 0.1 µg/mL ciprofloxacin, where no significant difference was observed relative to control.

It had been previously shown that the promoter of the PA3040 operon is under the control of the sigma factor AlgU, the master regulator of alginate production (32). AlgU is normally repressed by interaction with the anti-sigma factor MucA but is released in conditions of cell envelope stress, leading to alginate production as a protective response. In this study, it was proposed that PA3040 is dispensable in planktonic cells but that its absence causes cell envelope stress by an unknown mechanism in sessile growth conditions leading to constitutive AlgU-responsive gene transcription. Since our PA3040-3042 plasmid overexpression phenotype was consistent with involvement in regulation of cell surface polysaccharide production, we further examined the involvement of the operon in this pathway by investigating which conditions induced expression of its promoter. In general agreement with the previous study, we observed that the promoter was induced approximately fivefold from its very low basal level by cell envelope stress (acute D-cycloserine exposure in exponential phase wild-type cells) (Fig. 3A). We then challenged the promoter-reporter strain with acute phage exposure using our lytic phage ΦMSPA56 at MOI = 10 over a time course corresponding to one cycle of infection. Here, we observed that phage infection could also induce the promoter, as it increased during the time course to a significantly higher degree than the uninfected control, approaching similar levels to the D-cycloserine induction at the final (45 minutes) point of the time course (Fig. 3B). Therefore, the PA3040-3042 operon is also likely to be expressed as a response to lytic phage exposure, possibly as a result of the cell envelope damage caused by phage genome injection.

In order to investigate whether there was any autoregulation of P_PA3040_ upon overexpression or deletion of the genes, we transformed the promoter-reporter plasmid into the whole-operon deletion strain and the strains containing either the whole-operon overexpression plasmid or the empty vector control, and examined activity in exponential and stationary phases of growth. We did not observe any effect of deletion or overexpression of the operon on the activity of its own promoter, suggesting that even though it can feed back on activity of AlgU-dependent gene expression as measured by an *algD* promoter-reporter (32), it does not exert this effect on its own promoter. However, we found that activity of P_PA3040_ was very highly induced in stationary phase (Fig. 3C), far in excess of the D-cycloserine or lytic phage induction levels we observed in exponential phase cells. We also checked whether an unrelated stress (ciprofloxacin at inhibitory or sub-inhibitory concentrations) could induce the promoter and found that it did not do so (Fig. 3D). Therefore, we would anticipate that the gene products of the operon are barely produced at all in unstressed exponential planktonic growth but strongly expressed in stationary phase planktonic growth (despite not being identified in the *Pseudomonas aeruginosa* stationary phase sigma factor regulon [33] or the quorum-sensing regulon [34]). Taken together with our overexpression data (Fig. 2), these results support the hypothesis that this operon is part of the interconnected cell envelope stress and cell surface regulation networks and that failure to upregulate production of the PA3040-3042 genes could be a cause of the depletion of Tn insertions in PA3040 in the lytic phage infection condition of the Tn-Seq experiment, where high-MOI phage infection would likely cause substantial cell envelope stress.

### The PA3040-3042 operon does not affect bacteriophage resistance in pure culture assays in the PAO1 background but is required for normal growth of a clinical isolate of *P. aeruginosa*

**Fig 4:**
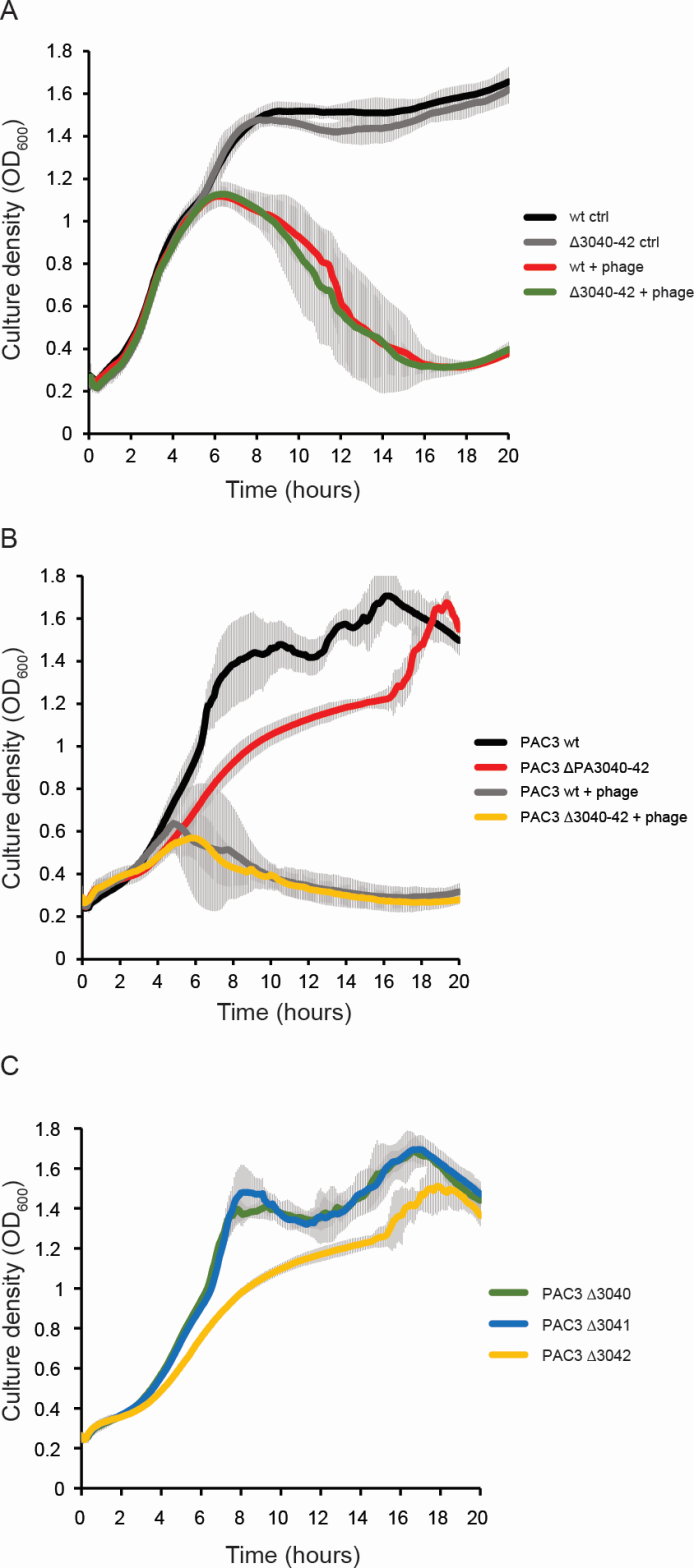
PA3040-3042 operon or PA3042 individual gene deletion causes a growth defect in a clinical isolate of *P. aeruginosa*. (**A**) Growth/death kinetics of WT PAO1 and deletion mutant of the PA3040-3042 operon, with or without ΦMSPA55 at MOI = 10^−7^. Error bars corresponding to standard deviation are shown for all data series (as gray overlay). (**B**) Growth/death kinetics of WT and deletion mutant of the PA3040-3042 operon in the *P. aeruginosa* clinical isolate PAC3, with or without ΦMSPA55 at MOI = 10^−7^. Error bars corresponding to standard deviation are shown for all data series as in panel **A**. (**C**) Growth kinetics of deletion mutants of the individual PA3040, PA3041, or PA3042 genes in the PAC3 isolate. Error bars corresponding to standard deviation are shown for all data series as in panel **A**.

Since the previous study examining the role of PA3040 had found that its loss of function (by transposon insertion) had led to constitutive σ^22^ activation under sessile growth conditions, we tested resistance to bacteriophage infection using our operon deletion and overexpression strains in the PAO1 background. However, we did not observe any effect of operon deletion or overexpression on resistance to either of our two lytic phages used in the Tn-Seq screen in spot plate assays (Fig. S1) or of the operon deletion strain to phage ΦMSPA55 in low-MOI phage killing curves in 96-well plate format (Fig. 4A). Experiments were set up identically to the growth curves of Fig. 2 but with phage ΦMSPA55 inoculated at an MOI of 10^−7^ so that the cell population would initially proliferate, then collapse once the phages had reached a sufficiently high concentration. In this setup, the control cultures without phage reached stationary phase at 8 hours after inoculation, while the phage-treated cultures grew with a similar profile to the control cultures until 6 hours, at which time they started to collapse, until the population was eliminated by 16–18 hours (Fig. 4A). We observed a slight tendency for the operon deletion strain to collapse faster than the wild type, consistent with the Tn-Seq results, but due to the very high level of variability between experiments, this was not statistically significant. The lack of effect of operon overexpression and deletion on sensitivity to lytic bacteriophage in either sessile or planktonic cells suggests that if the operon is involved in phage defense, it is likely to be redundant with the other phage defense systems in the genome.

Next, we deleted the PA3040-3042 operon in a clinical isolate of *Pseudomonas aeruginosa* (PAC3) obtained from an acute skin infection to investigate whether it influenced phage survival or other phenotypes in a strain that has not been laboratory adapted. In contrast to the PAO1 lab-conditioned strain, deleting this operon from the clinical isolate caused a growth defect in planktonic cells, with slower growth rate in exponential phase and initial stationary phase plateau reached at a lower OD_600_. This strain was also tested against the ΦMSPA55 phage, to which it is sensitive, but there was again high between-experiment variability and no significant difference in population collapse rate between the clinical isolate wild type and operon deletion strain (Fig. 4B). To investigate whether any particular gene from the operon was responsible for this growth defect, we deleted each of the three genes individually in PAC3 and repeated the growth experiment. Here, we observed that the growth defect was reproduced identically in the deletion of the third gene in the operon, PA3042, but not the other two (Fig. 4C). Therefore, it is most likely that this *P. aeruginosa*-specific uncharacterized gene is responsible for the defective growth phenotype, independently of the other two, and in a way which is specific to the clinical isolate background.

To compare this strain background against the PAO1 overexpression phenotype, we also overexpressed the operon in the PAC3 strain by streaking on IPTG plates alongside the empty vector. Here, we did not see any striking difference between empty vector and the overexpression construct, but in this case, it was because both strains produced small, regular-shaped round colonies, similar to PAO1 with the overexpression construct (Fig. S2A). Therefore, PAC3 may have different cell surface and/or extracellular polysaccharide characteristics to PAO1.

### PA3042 affects biofilm and pyocyanin production in the clinical isolate PAC3 but not PAO1

**Fig 5:**
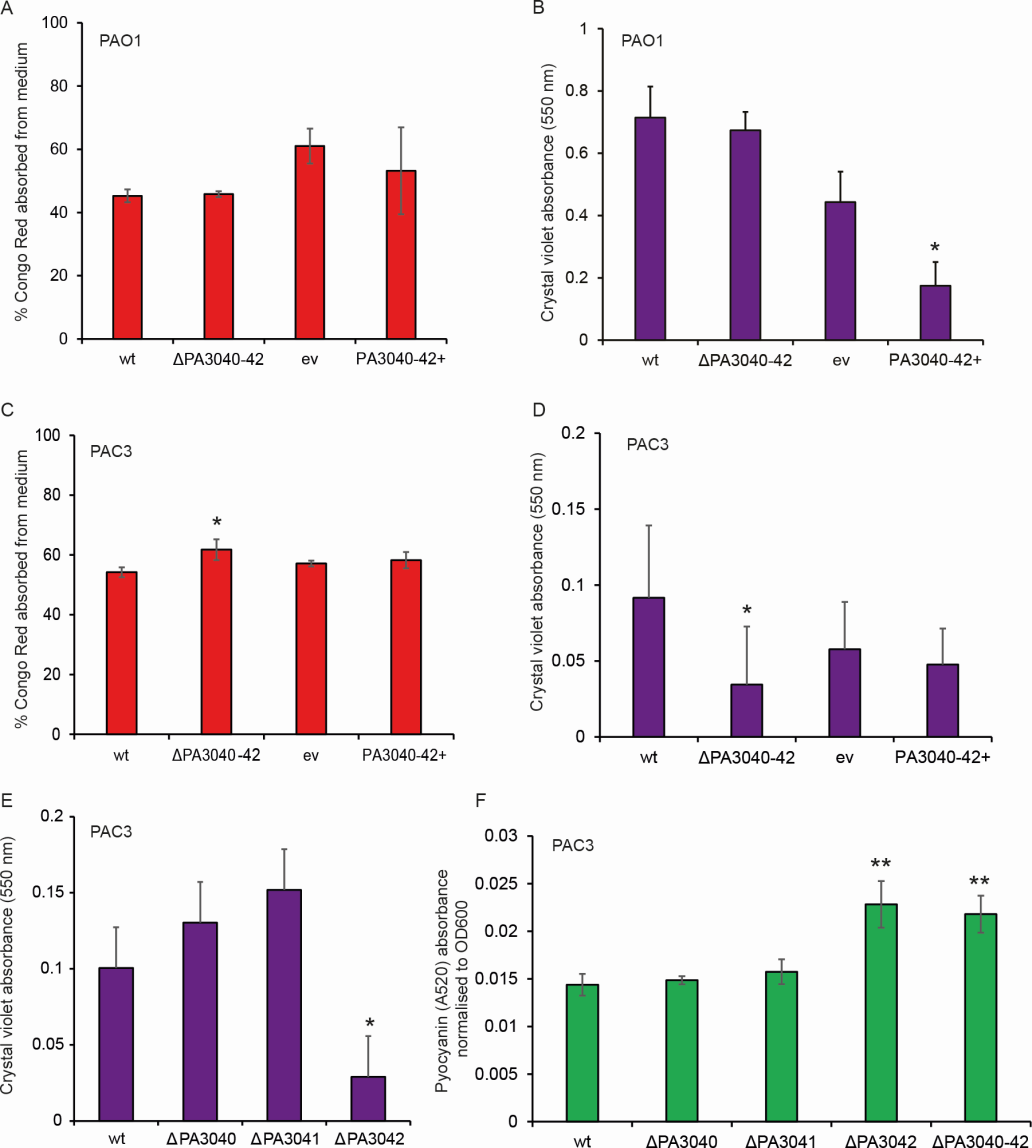
Deletion of the PA3040-3042 operon or PA3042 gene in the PAC3 clinical isolate, but not PAO1, affects biofilm formation and pyocyanin production. (**A**) Congo red absorption assay of WT PAO1, ΔPA3040-3042, WT containing empty pSRK::Gent (ev), and WT containing pSRK::PA3040-3042. (**B**) Crystal violet biofilm assay of WT PAO1, ΔPA3040-3042, WT containing empty pSRK::Gent (ev), and WT containing pSRK::PA3040-3042. The asterisk indicates the statistically significant difference (*P* < 0.05) between WT containing empty pSRK::Gent and WT containing pSRK::PA3040-3042. (**C**) Congo red absorption assay of WT PAC3, ΔPA3040-3042, WT containing empty pSRK::Gent (ev), and WT containing pSRK::PA3040-3042. The asterisk indicates the statistically significant difference (*P* < 0.05) between WT and ΔPA3040-3042. (**D**) Crystal violet biofilm assay of WT PAC3, ΔPA3040-3042, WT containing empty pSRK::Gent (ev), and WT containing pSRK::PA3040-3042. The asterisk indicates the statistically significant difference (*P* < 0.05) between WT and ΔPA3040-3042. (**E**) Crystal violet biofilm assay of WT PAC3, ΔPA3040, ΔPA3041, and ΔPA3042. The asterisk indicates the statistically significant difference (*P* < 0.05) between WT and ΔPA3042. (**F**) Pyocyanin estimation assay of WT PAC3, ΔPA3040, ΔPA3041, ΔPA3042, and ΔPA3040-3042. Asterisks indicate the statistically significant difference (*P* < 0.01) between WT and ΔPA3042 or ΔPA3040-3042 deletion strains.

Since we had observed changes in colony morphology of PAO1-overexpressing PA3040-3042 that were suggestive of changes in extracellular polysaccharide production (Fig. 2C), we investigated the effect of overexpression and deletion of these genes in both PAO1 and PAC3 strains on biofilm formation. Biofilm formation was tested by Congo red binding to cells grown in static liquid culture (Fig. 5A and C), as a measure of pellicle biofilm, and crystal violet staining of surface-adhered cells (Fig. 5B, D, and E). Surprisingly, these two assays did not give equivalent results. PAO1 cells had unchanged Congo red binding regardless of whether PA3040-3042 was deleted or overexpressed (Fig. 5A), while crystal violet staining was significantly reduced in the overexpression strain (Fig. 5B), suggesting that the altered colony morphology in this strain is correlated with reduced surface biofilm formation (but not necessarily with bulk polysaccharide production).

In the clinical isolate PAC3, Congo red binding was generally similar to that observed in PAO1 (Fig. 5C, compare to Fig. 5A) but very slightly increased in the operon deletion strain (Fig. 5C) and the PA3042 deletion strain (Fig. S2B). However, crystal violet staining was strongly reduced in WT PAC3 relative to WT PAO1 (Fig. 5D, compare vertical axis range to Fig. 5B) and reduced still further by operon deletion (Fig. 5D), while overexpression had no effect. Repeating this assay on the PAC3 isogenic strains with the individual genes deleted showed that the deletion of the PA3042 gene alone could reproduce the decreased crystal violet staining, while the other two genes of the operon did not affect it (Fig. 5E), similarly to what we had observed with the inhibited growth phenotype (Fig. 4C). Therefore, the PA3042 gene is required by the clinical isolate both for normal planktonic growth and for surface-associated biofilm production.

While performing these experiments, we had observed that overnight cultures of the operon deletion or PA3042 deletion strains in PAC3, although not PAO1, appeared noticeably greener than cultures of the isogenic WT. We therefore estimated whether pyocyanin production was altered, by measuring absorbance at 520 nm of culture supernatants and normalizing to OD_600_ to account for different culture densities between strains. Normalized A520 was significantly increased in the operon or PA3042 deletion strains (Fig. 5F), suggesting that these strains overproduce pyocyanin, in addition to their defects in growth and in biofilm production.

### PA3042 is a tobramycin resistance determinant in PAC3 but not PAO1

**Fig 6:**
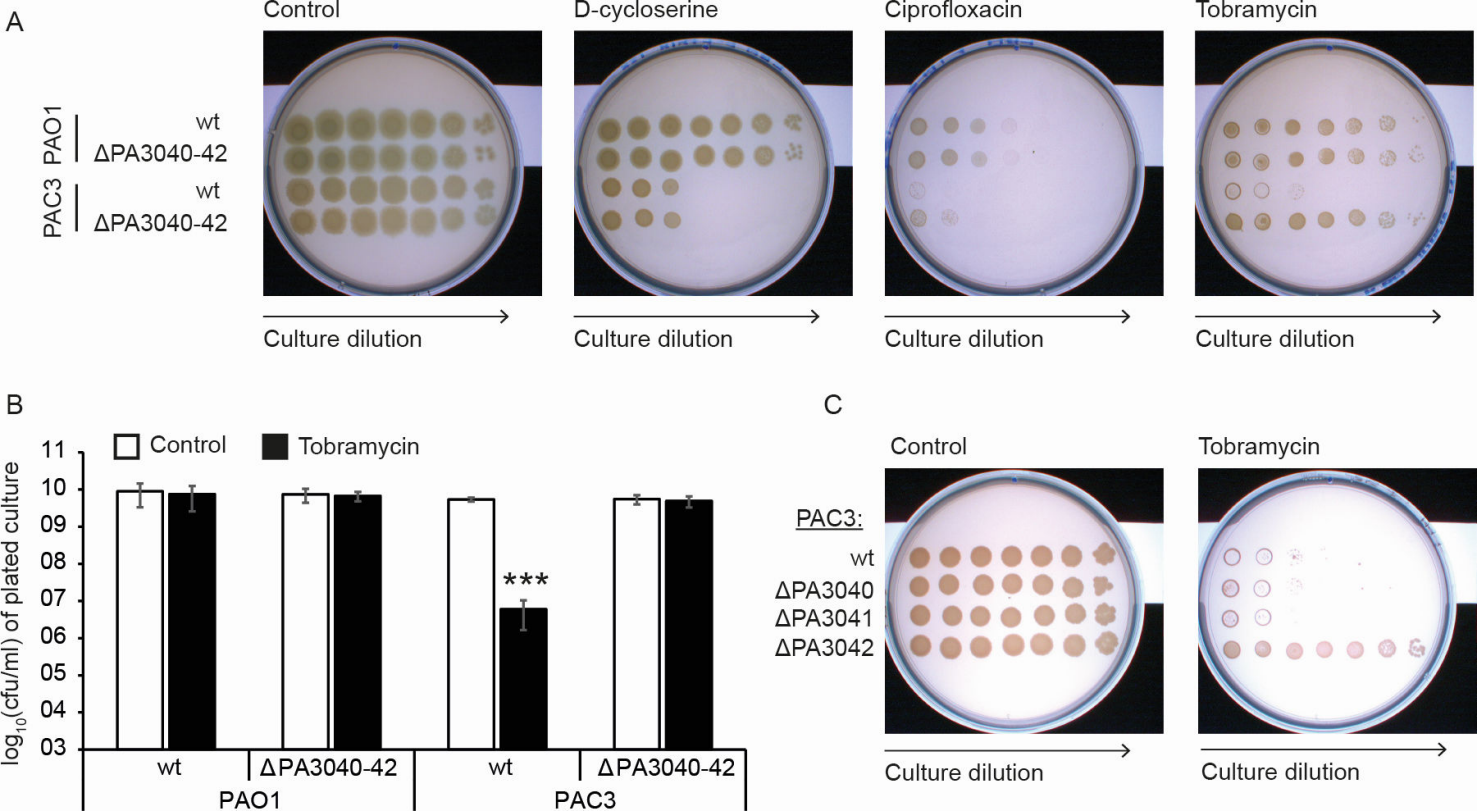
The PA3040-42 operon is involved in tobramycin sensitivity in PAC3 but not PAO1. (**A**) Efficiency of plating of PAO1 and PAC3 strains (WT and ΔPA3040-3042) on control plates, D-cycloserine (100 µg/mL), ciprofloxacin (0.05 µg/mL), and tobramycin (0.5 µg/mL). Images are representative of three biological replicates. (**B**) Quantification of cell viability on control and tobramycin plates of WT PAO1 and PAC3 and ΔPA3040-3042 deletion mutants in both backgrounds, from two technical and three biological replicates. Asterisks indicate the statistically significant difference (*P* < 0.001) between WT PAC3 and ΔPA3040-3042 in the presence of tobramycin. (**C**) Efficiency of plating of PAC3 WT and single-gene deletions of PA3040, PA3041, and PA3042 on control and 0.5 µg/mL tobramycin plates. Images are representative of three biological replicates.

Finally, we investigated whether any of the PA3040-3042 phenotypes were also associated with antibiotic resistance. We tested the sensitivity of PAO1 and PAC3 wild-type and PA3040-3042 operon deletion strains to D-cycloserine, ciprofloxacin, and tobramycin by the efficiency of plating assays (Fig. 6A). Although we had observed that D-cycloserine increased the activity of the operon’s promoter in PAO1 (Fig. 3A), the deletion strain in the PAO1 background did not have altered antibiotic sensitivity to this or either of the other two antibiotics. However, the PAC3 wild-type strain was noticeably more sensitive to all three antibiotics than PAO1 (Fig. 6A), especially tobramycin where viability was reduced by approximately three logs relative to PAO1 grown on tobramycin or PAC3 on control media (Fig. 6B). Deletion of the operon in the PAC3 background strongly increased tobramycin resistance to the level seen in PAO1 wild type (Fig. 6A and B). Similar to our observations with the biofilm and pyocyanin assays, we observed the same tobramycin resistance increase in the ΔPA3042 single-gene deletion (Fig. 6C), confirming that the PA3042 gene is responsible for it.

## DISCUSSION

In this study, we have initially focused on the PA3040-3042 operon of *P. aeruginosa* PAO1 as a candidate for a “backup” phage defense system, due to its appearance as one of the genes with significant depletion in lytic phage treatment compared to control, over all replicates of the infection experiments with the transposon insertion mutant library. Although the transposon read ratio was consistent with this function, we did not observe any evidence for the PA3040-3042 operon being a dedicated phage defense system since neither deletion nor overexpression of the operon affected phage resistance using the classical phage dilution spot assay in isogenic cultures. Experimental factors are probably responsible for this discrepancy. First, the transposon sequencing experiment is performed under unavoidable parameters that may influence the outcome relative to pure culture experiments. Proliferation of (cells containing) a certain transposon insertion is always measured as relative to all other transposon insertions in the pool, so the experiment may detect mutations that give a competitive advantage in growth, but for which the phenotype disappears in pure culture experiments where an isogenic mutant strain is compared to its parent strain grown separately. In addition, the transposon mutant pool is grown for the infection experiments under the extra stress of gentamicin in the medium to maintain selection on the transposon insertion, while it is absent from the experiments with the in-frame deletion mutants. Finally, we have found that this operon is highly growth phase dependent in its expression, so observation of differing phenotypes between wild-type and deletion mutant strains will be dependent on having the wild type in a phase of growth where the operon is actually expressed. Taking these considerations into account and noting that the normalized Tn insertion frequency into PA3040 is well above zero in both control and infection conditions (Data Set S1; Fig. 1B), we conclude that the most likely explanation is that our Tn-Seq screen has identified loci, including PA3040, that give a competitive disadvantage during growth under phage infection challenge relative to co-cultured cells with the loci undisrupted, but which do not have a strong enough effect on phage sensitivity for a disadvantage to be observed when comparing wild type to isogenic mutant cultured separately. A potential source for this competitive disadvantage could be that inability to upregulate the PA3040 operon upon phage exposure in mutants carrying the PA3040 Tn insertion leads to cell envelope changes that make these cells more infection susceptible than the others in the pooled culture. However, further experiments such as competition assays in the presence of phages or cell envelope-targeting agents would be required to test this hypothesis.

We observed increased PA3040 promoter activity after challenge with high MOI of lytic phage in early exponential phase. This is similar to the infection MOI of the transposon library, indicating that the PA3040 operon was likely to be actively transcribed in the infected mutant library. Seeing this response both in challenge with D-cycloserine and high-MOI phage infection, a shared mechanism could be considered. Simultaneous infection with a large number of phages can lead to “lysis from without,” which can be caused by the collective action of tail-associated lysozymes of multiple simultaneously adsorbing phages, as has been shown for T4 at MOI = 50 (35). While we did not observe premature culture lysis, non-lethal envelope damage could have engaged the cell envelope stress response akin to D-cycloserine and led to PA3040 induction. Links between phage infection and sigma factor-regulated envelope stress have also been established in other bacteria (36, 37). Investigation of functional category distribution among the significantly altered Tn insertion frequencies is also supportive of this possibility, since we observed that one of the most frequently seen gene categories among the depleted Tn insertions was cell envelope and membrane biogenesis (Table 1). Therefore, although we originally designed our screen to investigate phage sensitivity-related genes, the output thatwe have obtained from it seems more relevant to cell envelope stress and the list of depleted genes likely to contain other cell envelope stress response systems, rather than phage defense.

Paralogs of PA3040 and PA3041 have been identified in *E. coli* (*yqjD* and *yqjE*, respectively), and *yqjD* has been functionally characterized as a ribosome-binding membrane protein which contributes to ribosome inactivation as cells enter stationary phase (27). *yqjE* was identified in an overexpression screen for (p)ppGpp-independent persister cell formation as a toxic protein, which could increase persistence to antibiotics by drastically reducing growth rate (28). However, while both these *E. coli* proteins inhibit growth when individually overexpressed, we found that PA3040 and PA3041 do not do so, arguing for functional divergence between the *E. coli* and *P. aeruginosa* proteins. Instead, we found an overexpression phenotype to be (negatively) associated with biofilm formation and independent of growth rate in *P. aeruginosa* PAO1. Moreover, we only observed this phenotype when the operon was overexpressed and not upon overexpression of any of the individual genes, suggesting that all of the gene products of the operon must be present in order to inhibit biofilm formation.

Our data suggest that expression of this operon is most strongly induced by planktonic stationary phase in PAO1, more so than D-cycloserine or phage exposure, which argues for the primary regulator of this locus being growth phase and cell envelope stress being a secondary activator. While we have no data on whether P_PA3040_ expression is altered in sessile cells, the role proposed by Wood and Ohman for PA3040 in maintaining cell envelope homeostasis in sessile cells (32) would suggest that it should be expressed during sessile growth, including in the absence of cell envelope stress. However, in our assays investigating two modes of sessile growth (liquid-air interface pellicle, via Congo red binding, and surface biofilm, via crystal violet assay), we observed different phenotypes between our clinical isolates PAC3 and PAO1, both in wild type and in strains mutated for PA3040-3042. Both wild-type strains bound Congo red to a similar extent, suggesting similar abilities to make pellicle biofilm, but PAC3 had much lower crystal violet staining, suggesting reduced ability to form biofilms on surfaces relative to PAO1, and was much more sensitive to antibiotics (especially tobramycin). Moreover, PAC3 was the only strain in which we observed deletion phenotypes for these genes, specifically for PA3042. In the ΔPA3042 mutant, we found that growth rate was reduced in exponential phase, crystal violet staining was reduced even further than the PAC3 wild type, Congo red binding was slightly increased, pyocyanin production was increased, and tobramycin resistance was three logs higher than the PAC3 wild type. Taking the colony morphology, Congo red binding, and crystal violet assay results together, the most likely explanation is that PAC3 makes Pel polysaccharide but not Psl, permitting it to make pellicle biofilm but not effectively initiate biofilms on surfaces, as has been seen for the PA14 strain (38), while PAO1 makes both Pel and Psl (39–41). Considering that we observe deletion but not overexpression phenotypes in PAC3 but the inverse in PAO1, it is also possible that PAC3 expresses the operon at a higher level in exponential phase than PAO1 does, although we have not tested this. It is not clear whether these biofilm differences contribute to the tobramycin sensitivity and resistance that we see for the PAC3 wild type and ΔPA3042 mutant, respectively, but the improved resistance in the mutant could be linked to the increased pyocyanin levels. A recent study has shown that pyocyanin can induce tobramycin resistance by activating BrlR-dependent multidrug efflux pump expression (42).

In conclusion, we find that the PA3040-3042 operon, identified via a phage-sensitivity screen, is more likely to be a cell envelope stress and/or biofilm regulatory system. It appears to be important for normal growth and for antibiotic resistance in a clinical isolate of *P. aeruginosa*, but the lab-conditioned strain PAO1 may have adapted to do without it. The presence of the PA3042 gene in the *P. aeruginosa* species core genome, and the PA3040-3041 genes in the *Pseudomonas* genus core genome (with a few exceptions), argues for an important joint conserved function for these genes. As the current state of knowledge indicates that they are primarily associated with the stationary phase, biofilm, or sessile mode of growth (32), further investigation of these genes could clarify how cell envelope stress is tolerated in biofilms and/or in chronic infections, and whether their function would affect antibiotic resistance or bacteriophage exposure in this context.

## MATERIALS AND METHODS

### General growth conditions

Bacterial culturing was routinely done in Lennox LB broth (5 g/L yeast extract, 10 g/L tryptone, 5 g/L NaCl) at 37°C with shaking unless otherwise noted. Agar plates were made with growth medium supplemented with 1.5% agar. A top layer of LB broth and 0.5% agar was poured onto LB agar plates when making bacterial lawns. Plasmid selection and maintenance were done using antibiotics in the following concentrations: gentamicin at 50 µg/mL for *P. aeruginosa*, 10 µg/mL for *E. coli*; tetracycline at 75 µg/mL for *P. aeruginosa*, 10 µg/mL for *E. coli*. Overexpression of genes cloned into the pSRK::Gent vector (43) was induced by addition of 1 mM IPTG to cultures.

### Plasmid and strain construction

DNA fragments for cloning were amplified by PCR from *P. aeruginosa* PAO1 genomic DNA template, using Phusion polymerase (NEB) according to the manufacturer’s instructions, with specific primers listed in Table S2, and products were purified by agarose gel electrophoresis. Cloning of the correct region was confirmed by DNA sequencing, and plasmid stocks were cloned and maintained in TOP10 (Invitrogen). Plasmid characteristics are listed in Table S3.

For overexpression of PA3040 or the PA3040-3042 operon, genes were amplified with forward primer PA3040_SRK_fwd_v2 and reverse primers PA3040_SRK_rev (for the PA3040 gene) or PA3042_SRK_rev (for the operon), digested with *Nde*I and *Xba*I and ligated into correspondingly digested pSRK::Gent. For deletion constructs, upstream and downstream homology arms were amplified for each gene as detailed in Table S2 alongside the primer sequences. These consisted of 500–650 bp upstream and downstream regions of the gene to be deleted, including a small in-frame sequence corresponding to the protein termini of the gene. Upstream homology PCR products were digested with *Xho*I and *Bam*HI, while downstream homology PCR products were digested with *Bam*HI and *Hin*dIII. For each construct, the upstream and downstream PCR products were ligated simultaneously into *Xho*I/*Hin*dIII-digested pExG2. In order to construct the deletion construct for the whole operon, the upstream region of PA3040 (made with primers D_PA3040_A/B) and the downstream region of PA3042 (made with primers D_PA3042_C/D) were used. To construct the promoter-reporter construct, the promoter region of PA3040 was amplified using primers P3040_fwd_eco and P3040_rev_xba, digested with *Eco*RI and *Xba*I, and ligated into correspondingly digested plac290, a low-copy transcriptional fusion reporter plasmid.

pSRK::Gent and derivatives and the plac290::P_PA3040_ promoter-reporter plasmid were introduced into *P. aeruginosa* by electroporation. Cultures were washed twice in 300 mM sucrose and electroporated at 25 µF, 200 Ω, and 1.8 kV. Conjugation of suicide plasmids into *P. aeruginosa* was done using conjugable *E. coli* strains S17-1 *λpir* for pExG2 deletion constructs or SM10 *λpir* for pMR2xT7 transposon mutagenesis. Counterselection against conjugable *E. coli* was done by plating on M9 minimal medium (MgSO_4_ 2 mM, Na_2_HPO_4_ 42.2 mM, KH_2_PO_4_ 22 mM, NaCl 8.5 mM, NH_4_Cl 18.7 mM) supplemented with citrate (20 mM) and gentamicin. *P. aeruginosa* first integrant strains containing the deletion constructs integrated into the chromosome were subjected to sucrose counterselection to force deintegration of the plasmid by plating a mid-log culture on 0% NaCl 10% sucrose LBA plates. Plasmid deintegration was verified by plating on gentamicin plates, and successful deletion was verified by PCR. Full details of strains are listed in Table S4.

### Transposon mutant library construction

High-density transposon mutant libraries were generated using the pMR2xT7 suicide plasmid containing the MAR2xT7 transposon (44). We used our optimized protocol for high-efficiency conjugation from *E. coli* SM10 to PAO1. Briefly, this entails using early-stationary phase recipient and donor cells, mixing in a 3:1 donor:recipient ratio and washing twice in LB. Washed cells were resuspended in 1/10 of culture volume and spotted on LBA plates without antibiotics. The plate was incubated at 37°C for 6 hours and 15 minutes to conjugate. Spots were then resuspended in 1 mL 0.9% NaCl pre-warmed to 37°C and pelleted by centrifugation. A volume of 800 µL supernatant was removed, and pellet was resuspended in the remaining supernatant before plating on M9-citrate-gentamicin plates. Plates were then incubated at 37°C for 22 hours before transposon insertion mutants were harvested, pooled, and immediately stored in 10% glycerol at −80°C. A dilution series of the final plating suspension was used to approximate the number of mutants to 140,000 across 10 conjugations performed in parallel.

### Phage characteristics

Phage Fyn8 is a temperate phage isolated locally from river water (Odense, Denmark) and enriched on *P. aeruginosa* PAO1 (45). Phages ΦMSPA55 and ΦMSPA56 are virulent phages isolated from river water (Cambridge, UK) and enriched on two independent veterinary clinical isolates of *P. aeruginosa* (G. Salmond, personal communication).

### Infection experiments and transposon insertion sequencing

The pooled transposon library was passaged twice by pooling >10^8^ cells in LB medium supplemented with gentamicin and incubating overnight, then 1:50 diluting this culture into fresh medium and incubating overnight again. For the infection challenge, passaged cultures were diluted 1:50 into fresh medium and grown to mid-log phase (OD_600_ = approximately 0.6) before adding phage at an MOI of 10. Sampling was done 70 minutes post infection (approximately halfway through the lytic phage burst phase). Samples were immediately centrifuged at 14,000 g for 2 minutes. The supernatant containing phages and DNA of lysed bacteria was discarded, and genomic DNA was isolated from the pellet using Qiagen DNeasy Blood and Tissue Kit as specified by the manufacturer. Infection experiments were performed on three independent biological replicates.

Genomic DNA was prepared for Illumina sequencing using the Illumina DNA Prep Kit using customized PCR and primers to enrich for transposon junctions (Table S2). Forward primers consist of a transposon-specific adapter, Rd1 adapter, an i5 index for multiplexing, and P5 sequencing adapter. Reverse primers consist of Rd2 adapter, i7 index, and P7 sequencing adapter. PCR enrichment of the transposon junction sequence was done with Taq polymerase for 21 PCR cycles, the first four of these using an annealing temperature of 54°C and the remaining an annealing temperature of 66°C. Post-PCR purification was done with the bead-based purification specified in the manufacturer’s protocol using bead ratios of 0.4 for the right-side cleanup and 1.4 for left-side cleanup. Library quality and fragment size were verified using Agilent 5300 Fragment Analyzer, and sequencing was done on NovaSeq6000 and demultiplexed of indices and Illumina adapters, leaving the transposon-specific adapter. Overall sequencing quality was evaluated using FastQC 0.11.9. Transposon-specific adapter and transposon-originating sequences were trimmed from read 1 using Cutadapt 3.7 (46) while discarding untrimmed read pairs to remove gDNA reads and transposon adapters added unspecifically during PCR. Read pairs were then aligned to the PAO1 genome using Bowtie2 2.4.2 (47). Finally, Samtools 1.13 (48) was used to remove duplicate reads, before aligned reads were imported into Seqmonk 1.48.0 (49). Aligned reads were converted to per-gene counts, and genes with zero counts in either test or control conditions were filtered out before visualization and statistical analysis.

### Growth curves

ON cultures were diluted to give a starting OD_600_ of 0.2, and aliquots were transferred to a 96-well plate and incubated at 37°C for continuous OD_600_ measurements in a Synergy H1 plate reader (Biotek). When antibiotics or phages were used, these were added to the diluted ON culture. Growth curve cultures for the pSRK::Gent or inducible overexpression strains were supplemented with IPTG. Measurements were taken every 5 minutes for 20 hours. Experiments were performed in four technical replicates and three biological replicates, with average and standard deviation calculated from the biological replicates.

### Beta-galactosidase assay

Beta-galactosidase assay was performed by the method of Miller (50). In brief, ON cultures were subcultured 1:50 in fresh medium and grown to mid-log phase before applying the condition to be assayed. Culture was incubated at 37°C for 1 hour before OD_600_ was measured and β-galactosidase activity assayed. For the bacteriophage time course experiment, OD_600_ measurement and lysing were done at the time of sampling, whereafter samples were kept on ice until β-galactosidase activities could be assayed in parallel (immediately after the end of the time course). Experiments were performed in three biological replicates.

### Bacteriophage plaque assay

Lawns of the host strain to be assayed were prepared by casting 100 µL overnight culture into 6 mL molten top agar over LB base agar layer and solidifying at room temperature. Phage stocks were diluted to a starting concentration of 10^5^ pfu/mL, and a 10-fold dilution series of this was spotted (10 µL spots) on bacterial lawns. Spots were left to dry before incubating plates at 37°C for 16 hours. Images are representative of two independent biological replicates.

### Efficiency of plating assay

ON cultures of the strains to be tested were diluted to an OD_600_ of 0.5, which served as a starting point for 10-fold dilutions and plating of these on LB plates supplemented with antibiotics. Pictures were taken after 1 day of incubation at 37°C. Images are representative of three independent biological replicates.

### Quantification of efficiency of plating

To quantify previously observed antibiotic susceptibility, a whole-plate efficiency of plating assay was employed. ON cultures of the strains to be tested were diluted to an OD_600_ of 0.5 and then further diluted in a 10-fold dilution series. For control samples, 100 µL of a 10^−7^ dilution was spread on LBA plate without antibiotics. For test samples, 100 µL of an appropriate dilution for each strain was spread on LBA plates containing antibiotics. The appropriate dilution was chosen based on qualitative efficiency of plating experiments performed previously to obtain 20–200 colonies per plate. Plate counts were obtained from two technical replicates and three biological replicates of the experiments.

### Pyocyanin production estimation assay

Pyocyanin production differences between strains were estimated using crude culture supernatants. ON cultures were grown in LB media from colonies picked from agar plates. The ON culture was subcultured 1:100 and grown overnight. The cultures were measured for OD_600_, centrifuged at 14,000 g for 5 minutes, and the supernatant was collected for the measurement of OD_520_ (absorbance frequency of pyocyanin). The ratio between these was interpreted as an estimate of cell density-normalized pyocyanin production.

### Surface biofilm assay

Adherent biofilm production was assayed using a crystal violet-based method. ON cultures of the strains to be assayed were diluted 1:100 in growth medium and transferred to a 96-well plate and incubated at 37°C for 16 hours. Planktonic cells were then removed by firmly shaking the inverted plate and rinsing the plate by submersion twice. Biofilm was stained by 1.166× culture volume of 0.1% crystal violet to wells and incubated at room temperature for 10 minutes. Excess crystal violet was removed by shaking and rinsing in water. The plate was dried in fume hood until all water had evaporated. Stained biofilm was dissolved in 1.33× culture volume of 30% acetic acid and incubated at room temperature for 10 minutes. Endpoint measurement was OD_550_ of the dissolved biofilm. Experiments were performed in at least seven technical replicates and three biological replicates.

### Congo red binding assay

Exopolysaccharide production was assayed by growing strains in LB medium supplemented with 40 µg/mL Congo red. Media was not supplemented with gentamicin to maintain selection of plasmids with gentamicin resistance markers, as we observed gentamicin to cause Congo Red to precipitate out of solution. Cultures were grown at 37°C without shaking for 3 days to allow biofilm formation at the liquid-air interface. Samples were homogenized by vortexing and pipetting before centrifugation at 14,000 g for 5 minutes and OD_490_ measurement of supernatant. To adjust for background 490 nm absorbance by aqueous compounds in culture supernatant, a wild-type control culture without Congo red supplementation was subjected to same procedure and measurements, and OD_490_ of this was subtracted from sample OD_490_ measurements. This adjusted OD_490_ was subtracted from the OD_490_ of bacteria-free medium with Congo red to evaluate the percentage of Congo red absorbed by the biofilm.

### Statistical analysis

We statistically analyzed transposon sequencing results by converting reads to per-gene counts. Genes with statistically different counts between condition and control were identified using the EdgeR package, which uses a quantile-adjusted maximum likelihood to calculate maximum likelihood enrichment levels of each gene and statistically tests these between libraries using an exact test. False discovery correction was not employed. Statistical testing of beta-galactosidase reporter, biofilm, pyocyanin, and tobramycin resistance experiments was done using two-tailed, unpaired *t*-test between sample and the relevant control, as specified in the figure legends.

### Pangenome analysis

All 640 complete genomes of *Pseudomonas aeruginosa* were retrieved from the GenBank database (10 February 2023) along with the 113 representative genomes for the *Pseudomonas* genus (Table S1). Genomes were annotated using Prokka v1.14.6 at the standard parameters of the software (51). Two pangenomes were constructed, one for each genome collection using Roary v3.13.0 using the following settings: -e (multiFASTA alignment of core genes), -s (group paralogs), -ap (allow paralogs in core), and -cd 99 (core genes must be in 99% of genomes). For the species-wide pangenome, -i (blastp sequence identity for gene clustering) was set to 95%, and for the genus spanning pangenome, this was reduced to 90% (52). The Roary output files were analyzed by first identifying each gene of interest in the pangenome by blasting their reference sequence against the “pan_genome_reference.fa.” Gene frequency and distribution across the pangenomes were subsequently retrieved from the “gene_presence_absence.csv.”

Sequence conservation of the PA3040-PA3042 operon across both pangenomes was assessed by performing a tblastn of each translated gene sequence in the operon against all genomes using BLAST+. A match with an *E*-value cutoff at 10^−5^ was interpreted as a positive indicator of the selected genome harboring the gene, this was selected so as to only detect highly similar proteins. The relative position of each hit to the other members of the operon was also checked for each genome, with a hit only being considered a true operon member if it is within 1,000 bp of any other gene of the operon. Average percent sequence identity was calculated relative to the PAO1 gene sequences.

### COG category assignment

COG categories were assigned by submitting the sequences of all the genes found to be significantly differently enriched or depleted for Tn insertions in phage-infected conditions to eggNOG-mapper (eggnog-mapper.embl.de) with the default parameters of minimum hit *E* value of 0.001 and minimum 40% identity between query and subject.

## Data Availability

The Tn-Seq data have been deposited in GEO under accession number GSE246284.

## SUPPLEMENTAL MATERIAL

Supplemental material spectrum.03875-23-s0001.docx Fig. S1 and S2; Tables S1 to S4.

Supplemental data set spectrum.03875-23-s0002.xlsx Significantly depleted or enriched genes obtained from the phage infection Tn-Seq experiment.

ASM does not own the copyrights to Supplemental Material that may be linked to, or accessed through, an article. The authors have granted ASM a non-exclusive, world-wide license to publish the Supplemental Material files. Please contact the corresponding author directly for reuse.
