# Supplementary material for: The uncharacterized PA3040-3042 operon is part of the cell envelope stress response and a tobramycin resistance determinant in a clinical isolate of Pseudomonas aeruginosa
Source: Microbiol Spectr. 2024 Jul 1;12(8):e03875-23. doi: 10.1128/spectrum.03875-23 (PMC11302039; doi:10.1128/spectrum.03875-23)
Supplement: Supplemental material — Fig. S1 and S2; Tables S1 to S4. [file spectrum.03875-23-s0001.docx]

Supplementary material for:

***The uncharacterized PA3040-3042 operon is part of the cell envelope stress response and a tobramycin resistance determinant in a clinical isolate of Pseudomonas aeruginosa***

Contains:

Legend to Supplementary Dataset 1

Supplementary Figures S1 and S2

Supplementary Tables S1 to S4

**Supplementary Dataset legend**

***Supplementary Dataset 1. Significantly depleted or enriched genes obtained from the phage infection Tn-Seq experiment.*** Lists of genes in which Tn insertions were found to be significantly depleted or enriched in the phage infection conditions relative to control, alongside read counts for each condition, log2(fold change) and p values.

**Supplementary Figures**

**
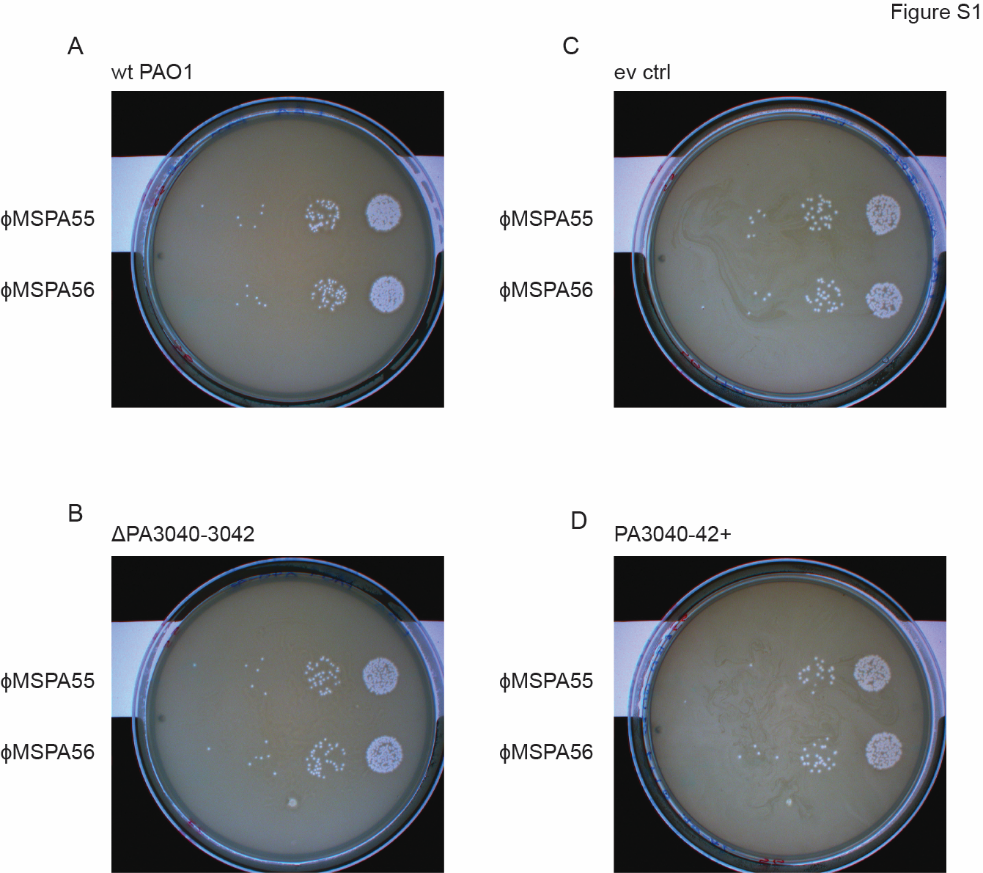
**

***Supplementary Figure S1. Neither deletion nor overexpression of the PA3040-3042 operon in PAO1 affects outcome of classical bacteriophage sensitivity assays.*** Plaque assays of the two lytic phages ΦMSPA55 and ΦMSPA56 on lawns of (A) WT PAO1, (B) ΔPA3040-3042, (C) WT containing pSRK::Gent empty vector and (D) WT containing pSRK::PA3040-3042. In C and D, agar was supplemented with gentamicin and 1 mM IPTG. Phages were spotted at approximate concentrations of 10^5^, 10^4^, 10^3^ and 10^2^ pfu/ml.


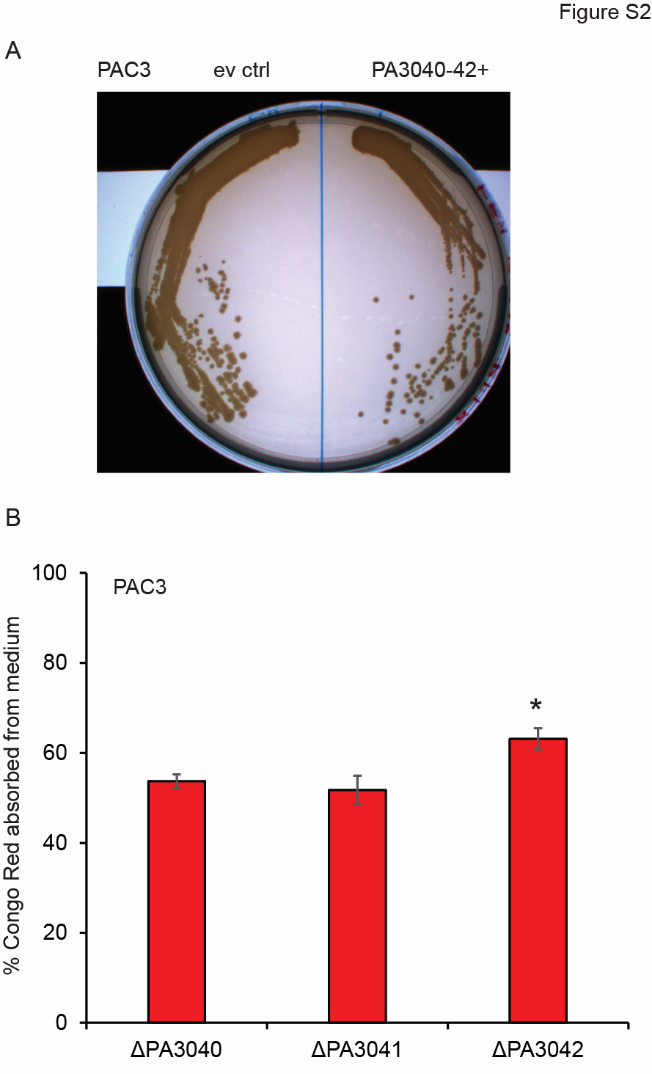


***Supplementary Figure S2. PAC3 has different colony morphology and biofilm properties than PAO1 upon overexpression of PA3040-3042 or deletion of PA3042.*** (A) Colony morphology of WT PAC3 containing either empty vector pSRK::Gent or the overexpression plasmid for the PA3040-3042 operon (pSRK::PA3040-3042), after growth on LB medium with 1mM IPTG for 16 hours. (B) Congo Red absorption assay of the single gene deletions of PA3040, PA3041 and PA3042 in the PAC3 strain. Statistical significance of the increase in the ΔPA3042 strain (p < 0.05) is calculated relative to the WT data shown in Figure 5C, since these were obtained from the same experiments.

***Table S1. Genomes used for construction of the Pseudomonas genus pangenome and presence/absence scores for the PA3040, PA3041 and PA3042 genes***

| **Accession** | **Genus** | **Species** | **Strain** | **PA3040** | **PA3041** | **PA3042** |
| --- | --- | --- | --- | --- | --- | --- |
| AE004091.2 | Pseudomonas | aeruginosa | PAO1 | 1 | 1 | 1 |
| AE016853.1 | Pseudomonas | syringae | tomato | 1 | 1 | 0 |
| CT573326.1 | Pseudomonas | entomophila | L48 | 1 | 1 | 0 |
| CP042804.1 | Pseudomonas | amygdali | tabaci | 1 | 1 | 0 |
| CP046035.1 | Pseudomonas | coronafaciens | oryzae | 1 | 1 | 0 |
| HG916826.1 | Pseudomonas | pseudoalcaligenes | 5344 | 1 | 1 | 0 |
| AP013070.1 | Pseudomonas | putida | 14164 | 1 | 1 | 0 |
| CP009365.1 | Pseudomonas | soli | SJ10 | 1 | 1 | 0 |
| CP007039.1 | Pseudomonas | cichorii | JBC1 | 1 | 1 | 0 |
| CP071706.1 | Pseudomonas | donghuensis | P482 | 1 | 1 | 0 |
| CP013124.1 | Pseudomonas | mendocina | S5.2 | 1 | 1 | 0 |
| CP009533.1 | Pseudomonas | rhizosphaerae | DSM | 1 | 1 | 0 |
| CP010896.1 | Pseudomonas | simiae | PCL1751 | 1 | 1 | 0 |
| CP012676.1 | Pseudomonas | versuta | L10.10 | 1 | 1 | 0 |
| CP014158.1 | Pseudomonas | citronellolis | P3B5 | 1 | 1 | 0 |
| CP014205.2 | Pseudomonas | glycinae | MS586 | 1 | 1 | 0 |
| CP014784.1 | Pseudomonas | alcaligenes | NEB | 0 | 0 | 0 |
| CP012400.2 | Pseudomonas | yamanorum | LBUM636 | 1 | 1 | 0 |
| CP014870.1 | Pseudomonas | silesiensis | A3 | 1 | 1 | 0 |
| CP014262.1 | Pseudomonas | corrugata | RM1-1-4 | 1 | 1 | 0 |
| CP015639.1 | Pseudomonas | lurida | L228 | 1 | 1 | 0 |
| CP017886.1 | Pseudomonas | frederiksbergensis | ERDD5:01 | 1 | 1 | 0 |
| CP018420.1 | Pseudomonas | veronii | R02 | 1 | 1 | 0 |
| AP014862.1 | Pseudomonas | furukawaii | complete | 1 | 1 | 0 |
| AP017423.2 | Pseudomonas | izuensis | complete | 1 | 1 | 0 |
| CP024159.1 | Pseudomonas | mosselii | PtA1 | 1 | 1 | 0 |
| CP020560.1 | Pseudomonas | paraeruginosa | Cr1 | 1 | 1 | 1 |
| CP029608.1 | Pseudomonas | kribbensis | 46-2 | 1 | 1 | 0 |
| CP031146.1 | Pseudomonas | plecoglossicida | XSDHY-P | 1 | 1 | 0 |
| CP032419.1 | Pseudomonas | cavernae | K2W31S-8 | 1 | 1 | 0 |
| CP022562.1 | Pseudomonas | monteilii | B5 | 1 | 1 | 0 |
| CP027706.1 | Pseudomonas | sessilinigenes | CMR12a | 1 | 1 | 0 |
| CP027756.1 | Pseudomonas | synxantha | R6-28-08 | 1 | 1 | 0 |
| CP027723.1 | Pseudomonas | orientalis | 8B | 1 | 1 | 0 |
| CP034338.1 | Pseudomonas | oryziphila | 1257 | 1 | 1 | 0 |
| CP026512.1 | Candidatus | Pseudomonas | isolate | 0 | 0 | 0 |
| CP035952.1 | Pseudomonas | tructae | SNU | 1 | 1 | 0 |
| CP035088.1 | Pseudomonas | viciae | 11K1 | 1 | 1 | 0 |
| CP034725.1 | Pseudomonas | brassicacearum | 3Re2-7 | 1 | 1 | 0 |
| CP044086.1 | Pseudomonas | luteola | FDAARGOS_637 | 0 | 0 | 0 |
| CP043311.1 | Pseudomonas | lalkuanensis | PE08 | 1 | 1 | 0 |
| CP046621.1 | Pseudomonas | alkylphenolica | Neo | 1 | 1 | 0 |
| CP047698.1 | Pseudomonas | knackmussii | N1-2 | 1 | 1 | 0 |
| CP049044.1 | Pseudomonas | psychrophila | KM02 | 1 | 1 | 0 |
| AP022642.1 | Pseudomonas | otitidis | DNA, | 1 | 1 | 0 |
| CP051487.1 | Pseudomonas | umsongensis | CY-1 | 1 | 1 | 0 |
| AP023189.1 | Pseudomonas | tohonis | DNA, | 1 | 1 | 0 |
| CP048833.1 | Pseudomonas | multiresinivorans | populi | 1 | 1 | 0 |
| CP053697.2 | Pseudomonas | campi | S1-A32-2 | 0 | 0 | 0 |
| CP054205.1 | Pseudomonas | rhodesiae | NL2019 | 1 | 1 | 0 |
| CP056030.1 | Pseudomonas | eucalypticola | NP-1 | 1 | 1 | 0 |
| CP060009.1 | Pseudomonas | sediminis | B10D7D | 1 | 1 | 0 |
| CP077084.1 | Pseudomonas | tritici | SWRI145 | 1 | 1 | 0 |
| CP077083.1 | Pseudomonas | salmasensis | SWRI126 | 1 | 1 | 0 |
| CP077089.1 | Pseudomonas | tensinigenes | ZA | 1 | 1 | 0 |
| CP077085.1 | Pseudomonas | shahriarae | SWRI52 | 1 | 1 | 0 |
| CP077090.1 | Pseudomonas | zeae | OE | 1 | 1 | 0 |
| CP077087.1 | Pseudomonas | monsensis | PGSB | 1 | 1 | 0 |
| CP077092.1 | Pseudomonas | iranensis | SWRI54 | 1 | 1 | 0 |
| CP077091.1 | Pseudomonas | hamedanensis | SWRI65 | 1 | 1 | 0 |
| CP077086.1 | Pseudomonas | zarinae | SWRI108 | 1 | 1 | 0 |
| CP077093.1 | Pseudomonas | vanderleydeniana | RW8P3 | 1 | 1 | 0 |
| CP077095.1 | Pseudomonas | xantholysinigenes | RW9S1A | 1 | 1 | 0 |
| CP077094.1 | Pseudomonas | promysalinigenes | RW10S1 | 1 | 1 | 0 |
| CP077097.1 | Pseudomonas | anuradhapurensis | RD8MR3 | 1 | 1 | 0 |
| CP061079.1 | Pseudomonas | chlororaphis | qlu-1 | 1 | 1 | 0 |
| CP062253.1 | Pseudomonas | gozinkensis | IzPS32d | 1 | 1 | 0 |
| CP062252.1 | Pseudomonas | allokribbensis | IzPS23 | 1 | 1 | 0 |
| CP063456.1 | Pseudomonas | shirazica | A28 | 1 | 1 | 0 |
| CP062158.2 | Pseudomonas | lundensis | 2T.2.5.2 | 1 | 1 | 0 |
| CP064354.1 | Pseudomonas | fragi | NL20W | 1 | 1 | 0 |
| CP067022.1 | Pseudomonas | cannabina | alisalensis | 1 | 1 | 0 |
| CP070503.1 | Pseudomonas | atacamensis | SM1 | 1 | 1 | 0 |
| CP070505.1 | Pseudomonas | toyotomiensis | SM2 | 1 | 1 | 0 |
| CP068551.1 | Pseudomonas | khazarica | ODT-83 | 1 | 1 | 0 |
| CP074578.1 | Pseudomonas | syringae | Susan2139 | 1 | 1 | 0 |
| CP074676.1 | Pseudomonas | qingdaonensis | S-1 | 1 | 1 | 0 |
| CP076668.1 | Pseudomonas | lijiangensis | LJ2 | 1 | 1 | 0 |
| CP077073.1 | Pseudomonas | muyukensis | COW39 | 1 | 1 | 0 |
| CP077077.1 | Pseudomonas | maumuensis | COW77 | 1 | 1 | 0 |
| CP077078.1 | Pseudomonas | azerbaijanorientalis | SWRI123 | 1 | 1 | 0 |
| CP077079.1 | Pseudomonas | asgharzadehiana | SWRI132 | 1 | 1 | 0 |
| CP077075.1 | Pseudomonas | xanthosomatis | COR54 | 1 | 1 | 0 |
| CP077076.1 | Pseudomonas | fakonensis | COW40 | 1 | 1 | 0 |
| CP077080.1 | Pseudomonas | alvandae | SWRI17 | 1 | 1 | 0 |
| CP071586.1 | Pseudomonas | germanica | FIT28 | 1 | 1 | 0 |
| CP081178.1 | Pseudomonas | mandelii | KGI_MA19 | 1 | 1 | 0 |
| CP076652.1 | Pseudomonas | savastanoi | MHT1 | 1 | 1 | 0 |
| CP075567.1 | Pseudomonas | fitomaticsae | FIT81 | 1 | 1 | 0 |
| CP072610.1 | Pseudomonas | wenzhouensis | A20 | 0 | 0 | 0 |
| CP128540.1 | Pseudomonas | alloputida | NMI24_14 | 1 | 1 | 0 |
| CP090029.1 | Pseudomonas | sivasensis | BsEB-1 | 1 | 1 | 0 |
| CP058908.1 | Pseudomonas | phenolilytica | RBPA9 | 0 | 0 | 0 |
| CP091088.1 | Pseudomonas | juntendi | PP_2463 | 1 | 1 | 0 |
| CP092923.1 | Pseudomonas | syringae | tagetis | 1 | 1 | 0 |
| CP095766.1 | Pseudomonas | chengduensis | T1624 | 1 | 1 | 0 |
| CP066270.1 | Pseudomonas | tremae | PA-1-10F | 1 | 1 | 0 |
| CP097108.1 | Pseudomonas | bijieensis | SP1 | 1 | 1 | 0 |
| CP099598.1 | Pseudomonas | siliginis | OTU6BANIB1 | 1 | 1 | 0 |
| CP078013.1 | Pseudomonas | pergaminensis | 1008 | 1 | 1 | 0 |
| CP100553.1 | Pseudomonas | hydrolytica | KHPS2 | 1 | 1 | 0 |
| CP101125.1 | Pseudomonas | nunensis | In5 | 1 | 1 | 0 |
| CP085684.1 | Pseudomonas | ficuserectae | ZJDX-003 | 1 | 1 | 0 |
| CP087185.1 | Pseudomonas | sichuanensis | B21-027 | 1 | 1 | 0 |
| CP102179.1 | Pseudomonas | canavaninivorans | B21-020 | 1 | 1 | 0 |
| AP023081.1 | Pseudomonas | solani | DNA, | 1 | 1 | 0 |
| CP119382.1 | Pseudomonas | kermanshahensis | Mr36 | 1 | 1 | 0 |
| CP120376.1 | Pseudomonas | nitroreducens | L4 | 1 | 1 | 0 |
| CP127871.1 | Pseudomonas | kurunegalensis | ANKC.G2 | 1 | 1 | 0 |
| CP130043.1 | Pseudomonas | kielensis | ZE23JCel16 | 1 | 1 | 0 |
| LT855380.1 | Pseudomonas | viridiflava | CFBP | 1 | 1 | 0 |
| LS999205.1 | Pseudomonas | protegens | genome | 1 | 1 | 0 |
| LR215729.2 | Pseudomonas | marincola | YSy11 | 0 | 0 | 0 |

***Table S2. Primers used in this study. Restriction enzyme sites used for cloning are underlined.***

| ***Name*** | ***Sequence (5’ – 3’)*** | ***Comments*** |
| --- | --- | --- |
| D_PA3040_A | AAACTCGAGAACAGGGTATTGGAGCCGTG | PA3040 upstream homology arm forward |
| D_PA3040_B | AAAGGATCCAGCGTTTGCAGTGGTCTTG | PA3040 upstream homology arm reverse |
| D_PA3040_C | AAAGGATCCCTGCTTGGCCTGTTGGTCA | PA3040 downstream homology arm forward |
| D_PA3040_D | AAAAAGCTTAACAGTTGCTGGCTGAGGTT | PA3040 upstream homology arm reverse |
| D_PA3041_A | AAACTCGAGGGGAATTGCAAGCGCCATAC | PA3041 upstream homology arm forward |
| D_PA3041_B | AAAGGATCCGTCCTTTCCTTCGCTCATGG | PA3041 upstream homology arm reverse |
| D_PA3041_C | AAAGGATCCGAAGAACTGGCCAACGACC | PA3041 downstream homology arm forward |
| D_PA3041_D | AAAAAGCTTTCATGGTCCTTGTGGAAGGC | PA3041 upstream homology arm reverse |
| D_PA3042_A | AAACTCGAGCAAGATCCACGACAGCCTCA | PA3042 upstream homology arm forward |
| D_PA3042_B | AAAGGATCCGTTCACCGGTAGTTGGCTCAT | PA3042 upstream homology arm reverse |
| D_PA3040-42_C | AAAGGATCCCGCAAGCAGAAAGGCCGTT | PA3042 downstream homology arm forward |
| D_PA3040-42_D | AAAAAGCTTGTGGAGAAAGTCGGAACGCT | PA3042 downstream homology arm reverse |
| PA3040_SRK_fwd_v2 | AAACATATGCCCCGCAAGACCACTGCA | PA3040 coding sequence forward |
| PA3040_SRK_rev | AAATCTAGATCAGCGCCGGCTGACC | PA3040 coding sequence reverse |
| PA3042_SRK_rev | AAATCTAGATTCAACGGCCTTTCTGCTTG | PA3042 coding sequence reverse |
| P3040_fwd_eco | AAAGAATTCAAGCTCCCGGACTTTCCTTG | PA3040 promoter forward |
| P3040_rev_xba | AAATCTAGAATCCTTTGCAGCGTTTGCAG | PA3040 promoter reverse |
| **Transposon sequencing library prep primers** | |  |
| **Forward primers** |  |  |
| CLK TnSEQ-01 | AATGATACGGCGACCACCGAGATCTACACCGAATACGTCGTCGGCAGCGTCAGATGTGTATAAGAGACAGGTCTAGAGACCGGGGACTTATCAGC |  |
| CLK TnSEQ-02 | AATGATACGGCGACCACCGAGATCTACACGTCCTTGATCGTCGGCAGCGTCAGATGTGTATAAGAGACAGGTCTAGAGACCGGGGACTTATCAGC |  |
| CLK TnSEQ-03 | AATGATACGGCGACCACCGAGATCTACACCAGTGCTTTCGTCGGCAGCGTCAGATGTGTATAAGAGACAGGTCTAGAGACCGGGGACTTATCAGC |  |
| CLK TnSEQ-04 | AATGATACGGCGACCACCGAGATCTACACTCCATTGCTCGTCGGCAGCGTCAGATGTGTATAAGAGACAGGTCTAGAGACCGGGGACTTATCAGC |  |
| CLK TnSEQ-05 | AATGATACGGCGACCACCGAGATCTACACGTCGATTGTCGTCGGCAGCGTCAGATGTGTATAAGAGACAGGTCTAGAGACCGGGGACTTATCAGC |  |
| CLK TnSEQ-06 | AATGATACGGCGACCACCGAGATCTACACATAACGCCTCGTCGGCAGCGTCAGATGTGTATAAGAGACAGGTCTAGAGACCGGGGACTTATCAGC |  |
| CLK TnSEQ-07 | AATGATACGGCGACCACCGAGATCTACACGCCTTAACTCGTCGGCAGCGTCAGATGTGTATAAGAGACAGGTCTAGAGACCGGGGACTTATCAGC |  |
| CLK TnSEQ-08 | AATGATACGGCGACCACCGAGATCTACACGGTATAGGTCGTCGGCAGCGTCAGATGTGTATAAGAGACAGGTCTAGAGACCGGGGACTTATCAGC |  |
| CLK TnSEQ-09 | AATGATACGGCGACCACCGAGATCTACACTCTAGGAGTCGTCGGCAGCGTCAGATGTGTATAAGAGACAGGTCTAGAGACCGGGGACTTATCAGC |  |
| CLK TnSEQ-10 | AATGATACGGCGACCACCGAGATCTACACTGCGTAACTCGTCGGCAGCGTCAGATGTGTATAAGAGACAGGTCTAGAGACCGGGGACTTATCAGC |  |
| CLK TnSEQ-11 | AATGATACGGCGACCACCGAGATCTACACCTTGCTAGTCGTCGGCAGCGTCAGATGTGTATAAGAGACAGGTCTAGAGACCGGGGACTTATCAGC |  |
| CLK TnSEQ-12 | AATGATACGGCGACCACCGAGATCTACACAGCGAGATTCGTCGGCAGCGTCAGATGTGTATAAGAGACAGGTCTAGAGACCGGGGACTTATCAGC |  |
| CLK TnSEQ-13 | AATGATACGGCGACCACCGAGATCTACACTATGGCACTCGTCGGCAGCGTCAGATGTGTATAAGAGACAGGTCTAGAGACCGGGGACTTATCAGC |  |
| CLK TnSEQ-14 | AATGATACGGCGACCACCGAGATCTACACGAATCACCTCGTCGGCAGCGTCAGATGTGTATAAGAGACAGGTCTAGAGACCGGGGACTTATCAGC |  |
| CLK TnSEQ-15 | AATGATACGGCGACCACCGAGATCTACACGTAAGGTGTCGTCGGCAGCGTCAGATGTGTATAAGAGACAGGTCTAGAGACCGGGGACTTATCAGC |  |
|  |  |  |
| **Reverse primers** |  |  |
| CLK TnSEQ-43bis | CAAGCAGAAGACGGCATACGAGATTTGCGAGAGTCTCGTGGGCTCGG |  |
| CLK TnSEQ-44bis | CAAGCAGAAGACGGCATACGAGATGAACGAAGGTCTCGTGGGCTCGG |  |
| CLK TnSEQ-45bis | CAAGCAGAAGACGGCATACGAGATCGAATTGCGTCTCGTGGGCTCGG |  |
| CLK TnSEQ-46bis | CAAGCAGAAGACGGCATACGAGATGGAAGAGAGTCTCGTGGGCTCGG |  |
| CLK TnSEQ-47bis | CAAGCAGAAGACGGCATACGAGATTCGGATTCGTCTCGTGGGCTCGG |  |
| CLK TnSEQ-48bis | CAAGCAGAAGACGGCATACGAGATCTGTACCAGTCTCGTGGGCTCGG |  |
| CLK TnSEQ-49bis | CAAGCAGAAGACGGCATACGAGATGAGAGTACGTCTCGTGGGCTCGG |  |
| CLK TnSEQ-50bis | CAAGCAGAAGACGGCATACGAGATTCTACGCAGTCTCGTGGGCTCGG |  |
| CLK TnSEQ-51bis | CAAGCAGAAGACGGCATACGAGATGCAATTCCGTCTCGTGGGCTCGG |  |
| CLK TnSEQ-52bis | CAAGCAGAAGACGGCATACGAGATCTCAGAAGGTCTCGTGGGCTCGG |  |
| CLK TnSEQ-53bis | CAAGCAGAAGACGGCATACGAGATGTCCTAAGGTCTCGTGGGCTCGG |  |
| CLK TnSEQ-54bis | CAAGCAGAAGACGGCATACGAGATGCGTTAGAGTCTCGTGGGCTCGG |  |
| CLK TnSEQ-55bis | CAAGCAGAAGACGGCATACGAGATCAAGGTACGTCTCGTGGGCTCGG |  |
| CLK TnSEQ-56bis | CAAGCAGAAGACGGCATACGAGATAGACCTTGGTCTCGTGGGCTCGG |  |
| CLK TnSEQ-57bis | CAAGCAGAAGACGGCATACGAGATGTCGTTACGTCTCGTGGGCTCGG |  |

***Table S3. Plasmids used and constructed in this study***

| ***Plasmid name*** | ***Description*** | ***Reference / source*** |
| --- | --- | --- |
| pMR2xT7 | λ*pir*-dependent ori, tra, AmpR, GentR, *himar1* transposon delivery vector | [40] |
| pExG2 | *gent^R^*, ColE1, *oriT*, *sacB*. Suicide vector with sacB counter-selectable marker for two-step allelic exchange | https://doi.org/10.1038/nprot.2015.115 |
| pExG2::ΔPA3040 | PA3040 deletion vector | This study |
| pExG2::ΔPA3041 | PA3041 deletion vector | This study |
| pExG2::ΔPA3042 | PA3042 deletion vector | This study |
| pExG2::ΔPA3040-42 | PA3040-42 deletion vector | This study |
| pSRK::Gent | *gent^R^*, *lacI*, P_lac_, IPTG-inducible expression vector | [39] |
| pSRK::PA3040 | PA3040 inducible overexpression vector | This study |
| pSRK::PA3040-42 | PA3040-42 inducible overexpression vector | This study |
| plac290 | oriV, Tet^R^, lacZ transcriptional fusion vector (low copy) |  |
| plac290::P_PA3040_ | PA3040 promoter-reporter construct | This study |

***Table S4. P. aeruginosa strains used and constructed in this study.***

| ***Strain genotype/name*** | ***Description*** |
| --- | --- |
| PAO1 | Wild type (wt) laboratory strain, source C. van Delden laboratory, University of Geneva |
| PAO1 ΔPA3040 | In-frame deletion of PA3040 |
| PAO1 ΔPA3040-42 | In-frame deletion of PA3040-3042 operon |
| PAO1 PA3040+ | PAO1 with pSRK::PA3040 |
| PAO1 PA3040-42+ | PAO1 with pSRK::PA3040-3042 |
| PAO1 pSRK::Gent | PAO1 with empty vector control for inducible overexpression constructs |
| PAO1 plac290::P_PA3040_ | PAO1 wt with PA3040 promoter-reporter |
| PAO1 ΔPA3040-42 plac290::P_PA3040_ | In-frame deletion of PA3040-3042 operon with PA3040 promoter-reporter |
| PAO1 PA3040-42+ plac290::P_PA3040_ | PAO1 with pSRK::PA3040-3042 inducible overexpression and PA3040 promoter-reporter |
| PAO1 pSRK::Gent plac290::P_PA3040_ | PAO1 with pSRK::Gm empty vector and PA3040 promoter-reporter |
| PAC3 | wt clinical isolate from acute wound infection, source Odense University Hospital |
| PAC3 ΔPA3040 | In-frame deletion of PA3040 in clinical isolate |
| PAC3 ΔPA3041 | In-frame deletion of PA3041 in clinical isolate |
| PAC3 ΔPA3042 | In-frame deletion of PA3042 in clinical isolate |
| PAC3 ΔPA3040-42 | In-frame deletion of PA3040-3042 in clinical isolate |
